# Strongly Coupled Morphological Features of Aortic Aneurysms Drive Intraluminal Thrombus

**DOI:** 10.1038/s41598-018-31637-6

**Published:** 2018-09-05

**Authors:** D. Bhagavan, P. Di Achille, J. D. Humphrey

**Affiliations:** 1 Department of Biomedical Engineering, Yale University, New Haven, CT USA; 2 Vascular Biology and Therapeutics Program, Yale School of Medicine, New Haven, CT USA

## Abstract

Over 75% of abdominal aortic aneurysms harbor an intraluminal thrombus, and increasing evidence suggests that biologically active thrombus contributes to the natural history of these potentially lethal lesions. Thrombus formation depends on the local hemodynamics, which in turn depends on morphological features of the aneurysm and near vasculature. We previously presented a hemodynamically motivated “thrombus formation potential” that predicts where and when thrombus might form. Herein, we combine detailed studies of the three-dimensional hemodynamics with methods of sparse grid collocation and interpolation via kriging to examine roles of five key morphological features of aneurysms on thrombus formation: lesion diameter, axial position, length, curvature, and renal artery position. Computational simulations suggest that maximum diameter is a key determinant of thrombogenicity, but other morphological features modulate this dependence. More distally located lesions tend to have a higher thrombus formation potential and shorter lesions tend to have a higher potential than longer lesions, given the same aneurysmal dilatation. Finally, movement of vortical structures through the infrarenal aorta and lesion can significantly affect thrombogenicity. Formation of intraluminal thrombus within an evolving abdominal aortic aneurysm thus depends on coupled morphological features, not all intuitive, and computational simulations can be useful for predicting thrombogenesis.

## Introduction

Abdominal aortic aneurysms (AAAs) are focal dilatations of the infrarenal aorta that are increasingly responsible for morbidity and mortality in our aging society. Over 75% of these lesions harbor an intraluminal thrombus (ILT) that tends to space-fill the dilated region of the aneurysm without encroaching on the original lumen. Notwithstanding some controversy, increasing evidence suggests that an ILT can play significant roles in the natural history of these lesions^1,2^, including contributions to potentially catastrophic rupture due to the release or activation of proteases that can locally weaken the aneurysmal wall^3,4^. Formation of an ILT depends on many factors, including concentrations of coagulation-associated plasma proteins. Another fundamental contributor to the formation and possible growth of thrombus within AAAs is the hemodynamics, which in turn depends strongly on morphological features of the lesion as well as the proximal and distal vasculature. Advances in medical imaging and computational methods now allow detailed patient-specific assessments of both lesion geometry and the associated hemodynamics, but general interpretations of such results are complicated by the extreme variability from patient-to-patient. Importantly, lesion characteristics often differ both in extent (e.g., different sizes and shapes) and in combination (e.g., a large diameter lesion coupled with either a proximal or distal location within the infrarenal aorta). It is extremely difficult, therefore, to identify key predisposing quantitative metrics or even to build reliable intuition given the myriad possible geometries and degrees and extents of intraluminal thrombus.

We previously showed using patient-specific computational models of the hemodynamics that a new phenomenological metric, the thrombus formation potential (*TFP*), can predict well where and when an ILT might form within an evolving AAA^5,6^. Briefly, this metric arose from the hypothesis that thrombus will form within the complex hemodynamic environment of an aneurysm if two conditions co-localize spatially and temporally: if the endothelium is rendered susceptible to thrombus formation due to a low time-averaged wall shear stress and a high oscillatory shear stress, and if this susceptible region is presented with shear-activated platelets. Conversely, computational studies of non-aneurysmal segments of the central vasculature that otherwise exhibit complex geometries and flows but not thrombus suggest that neither endothelial susceptibility nor presentation of activated platelets alone appears to initiate thrombus formation on an undamaged endothelium^5^. Herein, we use the *TFP* to evaluate how different combinations of five key morphological features – maximum lesion diameter, axial position of the lesion within the infrarenal aorta, projected lesion length, relative renal artery positions, and aortic curvature – contribute to the potential formation of a thrombus within an AAA. Toward this end, we introduce an analytical descriptor of idealized lesion geometries to facilitate systematic variations of the five morphological features and then perform 179 full unsteady 3-D hemodynamic simulations for those lesions that are identified as potentially revealing by an adaptive sparse grid collocation method. Then, using kriging, we interpolate results across this five-dimensional morphometric space to estimate thrombus formation more generally, thereby effectively extending significantly the number of lesions studied without necessitating the many additional computational simulations that would have been needed. In this way, one can more easily investigate parameter sensitivity and correlations among morphometric parameters, and thereby begin to build intuition otherwise not possible.

## Results

### Hemodynamic Predictions

**Figure 1:**
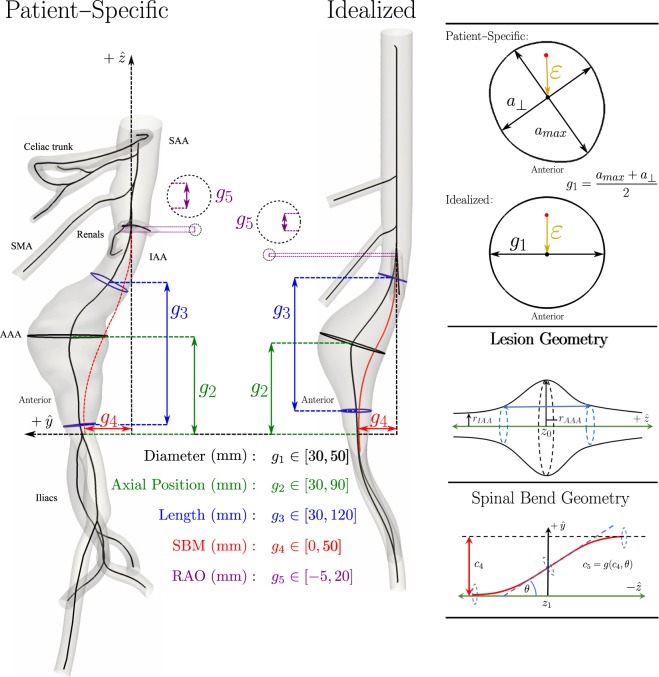
Parallel display of a reconstructed patient-specific AAA (left) and an idealized representation (right) that is defined geometrically by 5 constants (*c*_*i*_, i = 1, 2, …, 5); see text for the associated equations. Importantly, both lesions are characterized by 5 morphometric features: maximum diameter (*g*_1_), projected distance from the iliac bifurcation to the maximum diameter (*g*_2_), overall projected axial length (*g*_3_), a spinal bending magnitude (SBM or *g*_4_), and a renal artery offset (RAO or *g*_5_). A cross-section is shown for both geometries at the maximum diameter (top right) to highlight that parameter *g*_1_ is a mean value. The curvature and length of an idealized axisymmetric AAA (middle right) can be defined as a function of three constants (*c*_1_, *c*_2_, *c*_3_); for example, *c*_2_ defines the length of the lesion (*g*_3_). As seen in the lower right, the spinal bending magnitude (*g*_4_) is generated by two additional constants (*c*_4_ and *c*_5_). See text.

Figure 1 shows our simple yet broad parameterization scheme using five key geometric variables *g*_*i*_(*i* = 1 … 5) to characterize the morphology of each AAA: (1) the maximum diameter of the aneurysm (*g*_1_) is that value within the largest cross-section of the lesion orthogonal to the centerline, (2) the axial position of the aneurysm (*g*_2_) is the vertical distance from the iliac bifurcation to the geometric center of the largest cross-section, (3) the length of the aneurysm (*g*_3_) is the Euclidean distance between the proximal and distal entrances into the lesion, which are identified by a radius of the infrarenal abdominal aorta greater than 110% of the baseline value, (4) the spinal bending magnitude (SBM or *g*_4_) of the aneurysm is the maximum distance between the centerline of the lesion and the vertical axis of the aorta at the level of the iliac bifurcation, and (5) the renal artery offset (RAO or *g*_5_) is the signed distance between the ostia of the right and left renal arteries. These five parameters can be assessed from standard clinical imaging (e.g., computed tomography or magnetic resonance angiography) and are often considered by vascular surgeons when evaluating treatment options. An associated analytical representation (see Methods) enabled a straightforward generation of myriad idealized aneurysms suitable for systematic evaluation of the consequences of individual or combined morphological features.

**Figure 2:**
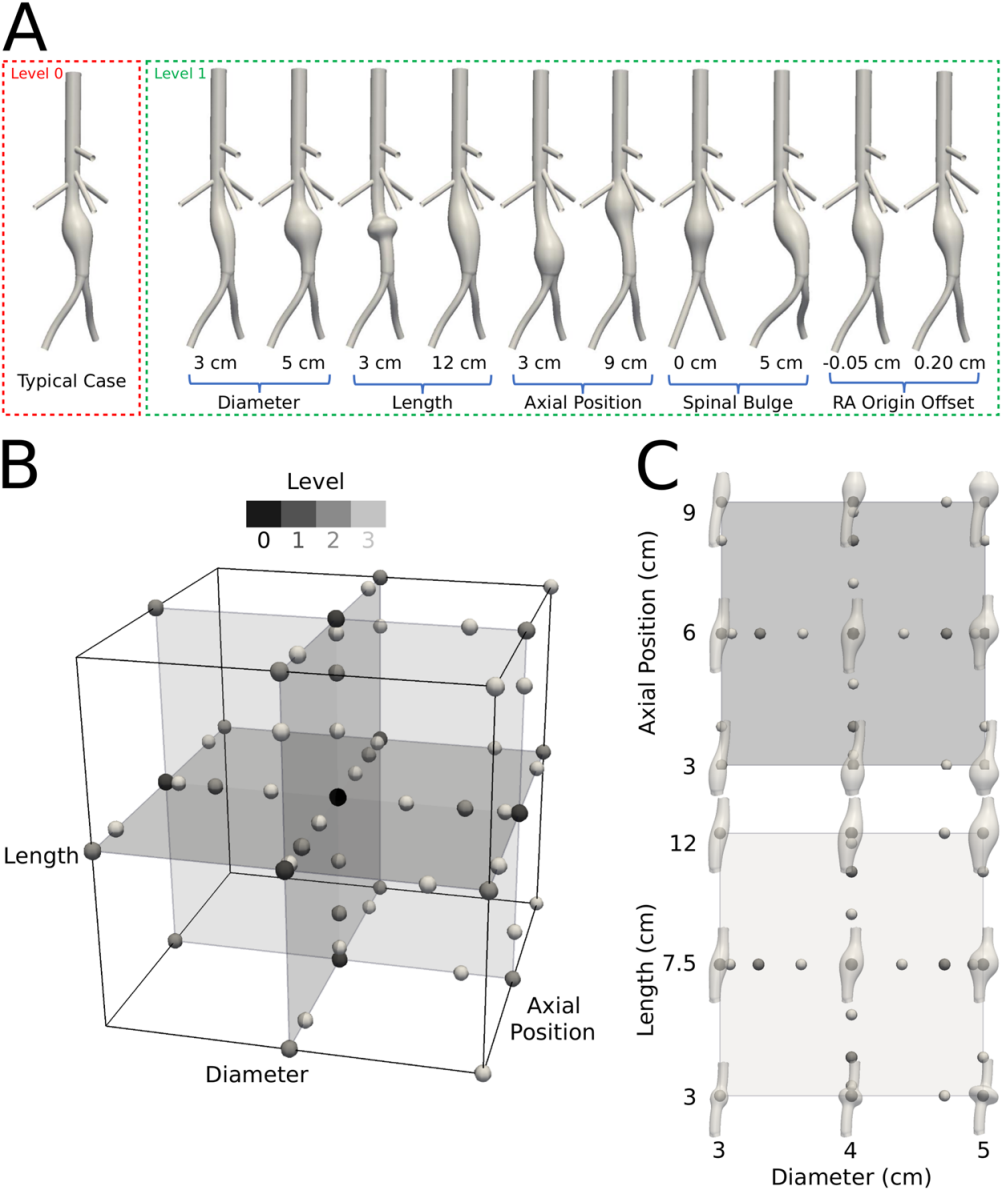
Illustration of the parameter space and sparse grid Chebyshev collocation scheme used to facilitate interpolation of hemodynamic metrics for the different geometries. (**A**) AAA geometries spanning the full ranges of the five explicit parameters *g*_*i*_. (**B**) 3-D projection of the 5-D parameter space that does not include variations in SBM and RAO, with vertices of the cube following a Chebyshev collocation scheme. Sparse grid nodes are grey-scale coded based on their level of refinement. (**C**) Anatomic variations on two mid-planes of the cube shown in B. The top plane shows combined variations in diameter and axial position of the lesion; the lower plane shows combined variations in diameter and length of the lesion. A total of 179 full hemodynamic simulations were performed (solutions of the Navier-Stokes equations plus Lagrangian particle tracking), results of which were used to interpolate via kriging across the entire parameter space.

Figure 2, panel A, shows a prototypical lesion geometry as well as morphologies that resulted from the extreme ranges of the parameters chosen (Table 1), noting that our focus was on thrombus formation and thus lesions early in their evolution. Shown, too, are representative “points” from our five-dimensional parameter space collapsed onto a three-dimensional space (panel B) to visualize multiple levels (0–3) of the collocation scheme that was used to identify those lesion geometries of most interest (see Methods). Finally, for further ease of visualization, we show select idealized geometries by superimposing a particular lesion geometry on the associated collocation point in two of the many possible two-dimensional projections (panel C). Overall, this adaptive collocation scheme suggested that we examine 179 individual lesions to evaluate how diverse hemodynamic and thrombus-related metrics varied as a function of changes in the five key morphological features.

**Table 1:** Dimensions and Bounds of Geometric Parameters used in Idealized Lesions.

Geometric parameter		Patient-Specific Measurements^5,6^	Experimental Bounds
Max Diameter (cm)	Mean ± SD	4.47 ± 0.54	4.00 ± 0.62
	Range	3.64–5.37	3.00–5.00
Axial Position (cm)	Mean ± SD	5.13 ± 1.37	6.00 ± 1.87
	Range	2.90–7.00	3.00–9.00
Length (cm)	Mean ± SD	5.88 ± 2.18	7.50 ± 2.80
	Range	3.07–9.00	3.00–12.00
Spinal Bending Magnitude (cm)	Mean ± SD	3.61 ± 1.03	2.50 ± 1.56
	Range	2.58–5.00	0.00–5.00
Renal Artery Offset (mm)	Mean ± SD	6.8 ± 6.5	7.5 ± 7.8
	Range	0.0–19.7	−5.0–20.0

**Figure 3:**
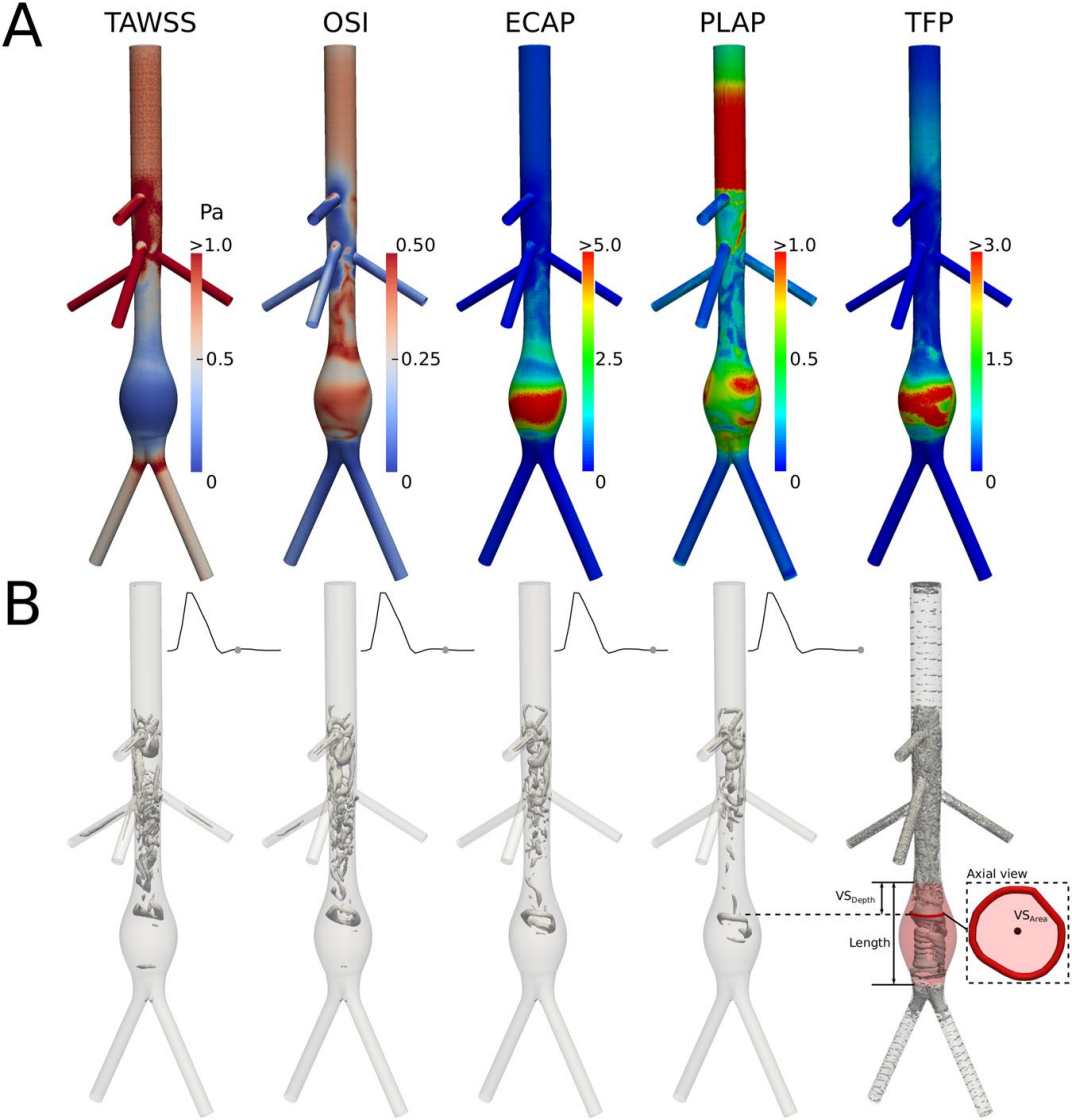
Useful hemodynamic and thrombogenicity indices illustrated for a single representative idealized lesion defined by: *g*_1_ = 4.0 cm, *g*_2_ = 3.0 cm, *g*_3_ = 7.5 cm, *g*_4_ = 0 cm, and *g*_5_ = 0.75 cm. (**A**) From left to right, time averaged wall shear stress (*TAWSS*), oscillatory shear index (*OSI*), endothelial cell activation potential (*ECAP*), platelet activation potential (*PLAP*), and most importantly thrombus formation potential *TFP*. *TAWSS*, *OSI*, and *ECAP* were determined directly by solving the 3-D unsteady Navier-Stokes equation (Eulerian formulation). *PLAP* was determined in post-processing using Lagrangian particle tracking, which allowed calculation of $TFP=ECAP\cdot PLAP$ at each point on the inner surface of the vessel. (**B**) From left to right, progressive evolution of *λ*_2_-isosurfaces (at *λ*_2_ = −200*s*^−1^) during diastole, showing formation and break-up of the main hairpin vortical structure (VS). On the bottom right, isosurfaces were constructed on the minimum *λ*_2_ field. From this, our analysis detects the location of the break-down of the VS (red contour), which allows computation of VS_Depth_, VS_Area_, and VS_Bending_ (the last one is not shown because it is equal to 0, as expected for this axisymmetric lesion).

Figure 3, panel A, illustrates key hemodynamic characteristics (e.g., distributions of wall shear stress (*WSS*), particle shear history, and vortical structures) via corresponding scalar metrics for a representative axisymmetric AAA (*g*_1_ = 4.0 cm, *g*_2_ = 3.0 cm, *g*_3_ = 7.5 cm, *g*_4_ = 0.0 cm, *g*_5_ = 0.75 cm) that has a high *TFP* (>3 within the lesion). Some features of the distributions of *WSS*-based indices seen in this AAA are common to most aneurysms. As expected, luminal enlargement reduces the time-averaged wall shear stress (*TAWSS*), particularly in central and distal regions of the lesion (e.g., 0.09 Pa when averaged over the central and distal lesion vs. 0.45 Pa when averaged over the healthy portion of the infrarenal aorta); see the first column in panel A. The oscillatory shear index (*OSI*) was high within the lesion in regions of low *TAWSS* (*OSI* = 0.33 averaged over the central and distal aspects) as well as in the healthy segment of the infrarenal aorta (*OSI* = 0.27) where the renal split induced oscillatory (i.e., backflow) flow during diastole (second column). Retrograde flow occurs in the infrarenal aorta in both healthy subjects and AAA patients, and is thought to favor atherogenesis and significantly influence AAA hemodynamics. Not surprisingly then, an Endothelial Cell Activation Potential (*ECAP*) index, which combines *OSI* and *TAWSS* after opportune scaling (see Methods), highlighted central and distal portions of the lesion as more likely to include susceptible endothelial cells (third column). Particle tracking revealed lower values of a platelet activation potential (*PLAP*) index, which accounts for the flow-induced shear history accumulated by blood-borne particles (see Methods), in the healthy portion of the infrarenal aorta (average value of 0.21), but higher values of *PLAP* throughout the AAA (average of 0.64), particularly on the sides of the proximal and distal necks of the lesion (average of 0.71 in those regions), as seen in the fourth column. These results suggest that lateral portions of the necks might collect flowing particles that have accumulated a significant shear history during their recent hemodynamic path, and may thereby convey activated platelets to the aneurysmal wall. The interplay between near-wall hemodynamics and particle advection is recapitulated by the distribution of *TFP* (rightmost column). The upper 99^th^ percentile of this index (denoted TFP-99p) for this lesion was 4.3, a value well above the thrombogenic threshold of 2.5 to 3.0 suggested by our previous patient-specific modeling that compared thrombogenic AAAs to non-thrombogenic carotid arteries and healthy infrarenal aortas^5^. *TFP* was high throughout the central part of the aneurysm, where *ECAP* was highest, and also laterally at the AAA necks, where *PLAP* was highest and *ECAP* indicated a potentially susceptible endothelium. In contrast, *TFP* was low everywhere else, suggesting that the combined occurrence of low and oscillatory *WSS* plus delivery of platelets having experienced a high shear history is peculiar to AAA hemodynamics, which renders the *TFP* index distinct among the metrics considered.

A more detailed interpretation of the distribution of multiple hemodynamic indices can be achieved by analyzing vortical structures through plots of negative λ_2_ iso-contours^7^. Figure 3, panel B, reveals a distinct hairpin vortical structure (VS) that originated during diastole at the start of retrograde flow within the infrarenal aorta. This structure was fully formed at about 80% of the time into diastole (i.e., 0.75 s into the 0.9 s cycle), but broke-up just before the end of the cardiac cycle. Small remnants of the larger VS remained throughout systole of the following beat, during which they were advected to the iliac bifurcation. While snapshots of systole are not shown directly, these events are discernable through iso-contour plots of the minimum λ_2_ field, which collects traces left by VSs throughout a cardiac cycle (see the rightmost column). Our simulations suggested that the position and geometry of VSs as they break-up differ significantly for different AAA geometries (not shown), which could help explain hemodynamic mechanisms underlying different values of *TFP* for different lesions. For the lesion highlighted in Fig. 3, break-up of the largest VS occurred proximally at about one-third of the aneurysm length (hence VS_Depth_ = 30%), and its area at break-up was 0.69 cm^2^, which corresponded to ~6% of the largest AAA cross-sectional area. As expected for an axisymmetric lesion with a zero SBM (i.e., *g*_4_ = 0.0 cm), the center of the VS aligned perfectly with the centerline of the lesion, thus VS_Bending_ was 0.

**Figure 4:**
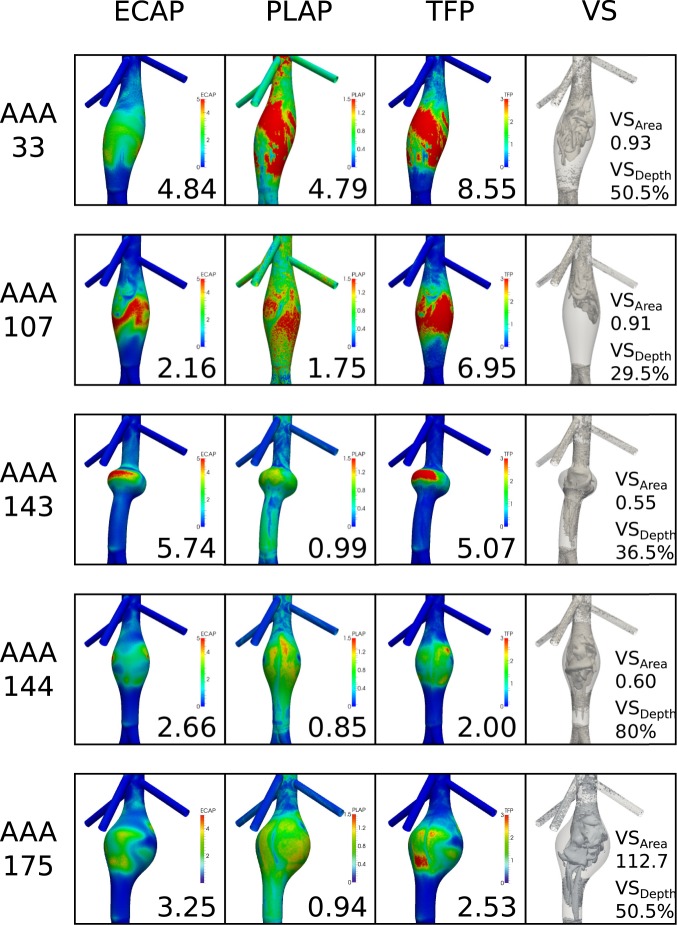
Illustrative results for five idealized models (of the 179 full models considered), showing 99^th^ percentile values of *ECAP*, *PLAP*, and *TFP* as well as VS_Area_ and VS_Depth_, indices related to vortical structures (cf. Fig. 3). These cases were selected given the a priori non-intuitive findings. See text for details but note that [*g*_1_, *g*_2_, *g*_3_, *g*_4_, *g*_5_] = [4, 6, 12, 2.5, 2.0], [4, 6, 12, 0, −0.5], [4, 7.0607, 3, 2.5, 0.75], [4, 7.0607, 7.5, 0, 0.75], and [5, 7.0607, 7.5, 2.5, 0.75], for lesions 33, 107, 143, 144, and 145, respectively.

In contrast, Fig. 4 shows five lesions (selected from the 179 full simulations) that exhibited some unexpected values of the thrombogenic indices. Together, these AAAs illustrate, in part, why hemodynamic simulations can be helpful in predicting AAA thrombogenicity, particularly in cases that cannot otherwise be intuited or explained by simple correlations to individual morphometric features of an aneurysm. The first two rows of Fig. 4 show 4-cm diameter lesions (i.e., *g*_1_ = 4 cm) that are positioned centrally within the infrarenal aorta (*g*_2_ = 6 cm) and have the maximum allowed length (*g*_3_ = 12 cm). The first lesion, AAA 33 (1^st^ row), had a mid-range value of spinal bending (*g*_4_ = 2.5 cm) but a maximum value of renal artery offset (*g*_5_ = 2.0 cm); we thus denote this lesion by ${\bar{g}}_{33}=[4,\,6,\,12,\,2.5,\,2.0]$. This lesion was selected because it showed the highest upper percentile values of *PLAP* and *TFP* (4.79 and 8.55, respectively) among all idealized aneurysms considered. For reference, mean values of *ECAP*, *PLAP*, and *TFP* were 3.98, 1.01, and 3.46, respectively, for all lesions having a 4.0 cm diameter; the proposed *TFP* threshold of 2.5 to 3.0 would thus suggest that 4.0-cm diameter lesions would be weakly thrombogenic on average. Moreover, given the low diameter-to-length ratio (4:12), one might not have expected high thrombogenicity in AAA 33 despite its large lesion volume, and given its slight tortuosity one might have expected flow stagnation (and therefore higher *TFP*) antero-laterally. Yet, the peculiar coupling of morphologic features in this AAA generated pronounced flow mixing and a heterogenous ensemble of VSs of varying sizes, most of which broke-up just below the axial center (VS_Depth_ = 51%). As for all lesions with a forward SBM (*g*_4_ = 2.5 cm), the VSs shifted towards the back of the aneurysm close to the posterior wall (VS_Bending_ = 0.77 cm). *ECAP* was thus higher towards the front of the aneurysm, and high *PLAP* manifested across the lesion wall, resulting in high *TFP* regions on the anterior proximal neck, anterior center of the AAA, and lateral and posterior portions of the distal neck.

AAA 107 (2^nd^ row) was fully axisymmetric (*g*_4 = _0 cm) and had a small RAO (*g*_5_ = −0.5 cm), but otherwise the same maximum diameter, axial position, and overall length as AAA 33; namely, ${\bar{g}}_{107}=[4,6,12,0,-0.5]$. The lack of tortuosity suggests that the only asymmetrical bias would arise due to the RAO, which was minimal. This different combination of features again resulted in pronounced mixing, with high *ECAP* occurring only at the anterior center of the lesion while *PLAP* was high almost everywhere within the central region of the lesion. The largest VSs broke-up proximally (VS_Depth_ = 29.5% of aneurysm length), releasing high shear history particles that were advected to central and distal portions of the lesion (i.e. where *PLAP* was highest) during the following systole. *TFP* was again high on the anterior surface and, with a TFP-99p of 6.95, this lesion had high thrombogenic potential.

The 3^rd^ row of Fig. 4 shows AAA 143, ${\bar{g}}_{143}=[4,7.0607,3,2.5,0.75]$. It is thus a much shorter lesion (*g*_3_ = 3 cm) though with the same diameter (*g*_1_ = 4 cm) and a more proximal location (*g*_2_ = 7.1 cm) with a moderate SBM (*g*_4_ = 2.5 cm). This lesion has a small volume, so one might anticipate a low *TFP*. Due to the tortuosity, one might expect flow stagnation (and therefore higher *TFP*) to occur antero-laterally. Due to the high axial position and immediately proximal branching vessels, one might expect disturbed flows and therefore high *TAWSS* and low *TFP*. AAA 143 showed high *ECAP* values, however, driving high thrombogenicity (upper percentile values of 5.74 and 5.07 for *ECAP* and *TFP*, respectively). Minimum λ_2_ iso-contours (rightmost column of 3^rd^ row) suggest that a high aspect ratio can induce VSs that do not deviate significantly from their pre-lesion path, resulting in lower *TAWSS*. In this case, spinal bending (SBM) exacerbated this effect on the anterior portion of the lesion, which showed the highest *ECAP* and *PLAP*, with a TFP-99p well above the expected threshold.

Finally, the last two rows of Fig. 4 show counter-intuitive examples for two larger AAAs (*g*_1_ = 4.0 cm and 5.0 cm, respectively, 4^th^ and 5^th^ rows) with thrombogenic indices that are either lower than or on the lower end of our proposed threshold for *TFP*. The fourth row depicts AAA 144, ${\bar{g}}_{144}=[4,7.06,7.5,0,0.75]$, a more proximally-located lesion having a standard diameter, length, and RAO, but no tortuosity. This lesion thus has a typical volume and aspect ratio, which might naively suggest a more “typical” TFP presentation; recall that the average TFP-99p for lesions with a 4.0-cm diameter and 7.5-cm length was 3.10, so one might expect this lesion to be thrombogenic. The lack of tortuosity implies that the only asymmetric bias would be due to RAO, which was mid-range, so one might expect biasing of high TFP to the left side. Yet, this lesion highlights how a proximal axial positioning can lower *TFP*, a recurring finding of our parametric study. In particular, AAAs located closer to the renal bifurcation tend to be exposed to higher shear stress (as expected near a flow discontinuity), and can be traversed by heterogeneous vortical structures that often maintain their integrity to the distal neck of the lesion. Indeed, for AAA 143 (3^rd^ row), VS break-up was close to the distal neck (VS_Depth_ = 80%), well beyond the high *ECAP* islet on the front side of the proximal neck. Not surprisingly, then, with little overlap of *ECAP* and *PLAP*, AAA 144 (4^th^ row) had an upper percentile *TFP* of only 2.0, below our proposed threshold for thrombogenicity.

The lesion in the 5^th^ row, AAA 175, ${\bar{g}}_{175}=[5,7.0607,7.5,2.5,0.75]$, had the same axial position as AAA 144, but maximum diameter and spinal bending within the range considered. From diameter alone, one might expect this lesion to be highly thrombogenic (high TFP-99p); due to the high aspect ratio, one might also expect greater flow stagnation and thus higher TFP. Yet, the more proximal axial position lowered *ECAP *and thus *TFP*, and because of spinal bending, the VSs tended to remain in the posterior half of the lesion (rightmost column of the 5^th^ row) before breaking-up at about 50% of the aneurysm length. Plots of *TFP* suggest that particles released by the breaking-up of a VS may find a susceptible endothelium on the anterior distal neck, where *ECAP* was highest (with an upper percentile value of 3.25). The relatively far distance between the high *ECAP* region and VS break-up could explain a TFP-99p of only 2.53, which was lowest among all lesions with a maximum diameter of 5.0 cm. Taken together, then, Fig. 4 suggests again that it need not be a single dominant morphological feature that dictates where and when a thrombus will form.

### Statistical Inference

**Figure 5:**
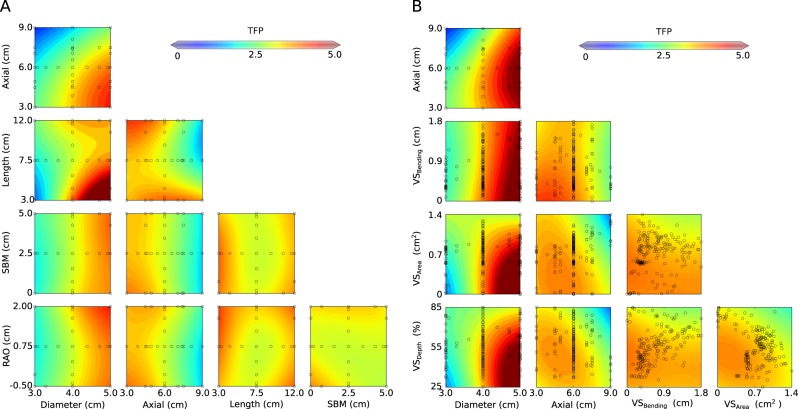
Interpolated (using kriging based on the 179 full simulations) values of the maximum predicted thrombus formation potential (*TFP*) shown in multiple 2-D projections from the 5-D parameter search space (left – axial position, projected axial length, SBM, and RAO plotted versus maximum diameter, axial position, projected length, and SBM as well as for multiple measures of vortical structure (right – axial position, VS_Bending_, VS_Area_, VS_Depth_ vs. maximum diameter, axial position, VS_Bending_, and VS_Area_). The open black circles show explicit evaluation points (of the 179 total) based on the sparse grid collocation. Note, for example, that the largest values of *TFP* were found for distally located, large diameter, and short axial length lesions.

Although it is useful to consider individual cases, as in large patient-specific data sets, it can be more revealing to systematically assess the influence of each geometric parameter on thrombogenicity while determining possible coupling amongst the parameters. Figure 5, panel A, depicts combinatorial maps of TFP-99p for metrics of geometry, hemodynamics, and vortical structures. Kriging was used to interpolate TFP-99p results from the 179 full simulations over the five-dimensional space Ξ formed by *g*_*i*_ over the specified ranges (see Methods and Supplement). Panel A shows 2D slices of Ξ for TFP-99p resulting from multiple combinations of two of the *g*_*i*_, with all other parameters fixed at their median values within each slice. Results from the 179 full simulations that resided in that plane are depicted by open circles. Panel B shows similar maps, except among diameter (*g*_1_), axial position (*g*_2_), VS_Bending_, VS_Area_, and VS_Depth_. These parameters are most responsible, in descending order (40%, 30%, 10%, 10%, 10%, respectively), for variability in TFP-99p, as determined by ANOVA of the 179 simulations. Panel 5B does not show slices of Ξ, but rather similar slices of another parametric space, Ξ', composed of this particular set of parameters. All 179 points are projected onto each slice in this case.

The first column of Fig. 5A relates lesion diameter (*g*_1_-abscissa) to the other four morphological features (*g*_2−5_-ordinates). Effects of maximum diameter tend to combine with the axial position (*g*_2_-1^st^ row) and length (*g*_3_-2^nd^ row) of a lesion to increase thrombogenic potential (i.e., TFP-99p > 2.5), but less so with SBM (*g*_4_-3^rd^ row) and RAO (*g*_5_-5^th^ row). Consider first the first row/first column (maximum diameter and axial position). While TFP-99p tends to increase monotonically with diameter, the gradient increases as the lesion location becomes more distal. Importantly, this coupling again suggests that more proximal lesions may be protected from thrombogenesis if the maximum diameter is 5 cm or less, with smaller lesions having a more distal location also appearing to be less susceptible to thrombogenesis. Results in the second row/first column show combined effects of maximum diameter and axial length, which are highly nonlinear with several regions of interest. TFP-99p again tends to increase monotonically with diameter, but the effect of length on this gradient is not monotonic. One region of interest is the lower right of the map. These lesions have a large diameter and short length (high aspect ratio) and are extremely thrombogenic, tending to have TFP-99p > 5. Indeed, AAA 143, depicted in the 3^rd^ row of Fig. 4, falls within this region. A second region of interest is the upper right of the map. These lesions have a large diameter and length (large volume) and tend to be thrombogenic, though not as thrombogenic as those with a high aspect ratio. Interestingly, there is a valley between these two regions where the Gaussian curvature is negative, approaching zero (recall that these maps are projections onto a plane for easier visualization). This implies that for large diameters, particular aspect ratios may be more protective against thrombus, especially at certain axial positions. This behavior is not seen at small diameters. The third row/first column shows coupling between maximum diameter and SBM, which yields minimal to modest effects on TFP-99p, hence this map primarily shows the effect of diameter. The fourth row/first column shows coupling between maximum diameter and RAO, which again yields small TFP-99p in many regions though slightly higher thrombogenicity at large diameters and large renal artery offsets. Interestingly, the gradient with diameter appears most right-shifted when RAO is zero and the geometry is bilaterally symmetric, suggesting that this state may be more protective against thrombus.

The second column of Fig. 5A relates effects of axial position (*g*_2_-abscissa) with three other *g*_*i*_ (ordinate) metrics (see the first row/first column for its relation to maximum diameter). As noted before, more proximal lesions (i.e., those a greater distance from the iliac bifurcation) tend to have lower thrombogenicity, and below a certain axial position, the behavior and coupling of other parameters appear to dominate. The second row/second column shows coupling between axial position and lesion length, which is highly nonlinear. Short lesions (*g*_3_ < 5) have high values of TFP-99p that appear to be relatively insensitive to axial position, whereas a saddle point appears to develop at moderate axial position and length, again implying that a certain aspect ratio (diameter is fixed at 4.0 cm in this slice) may be protective against thrombus. Longer lesions tend to be thrombogenic only if located more distally. The third row/second column shows coupling between axial position and SBM, which appears to be minimal, with a slight rightward shift of the axial position TFP-99p gradient for lesions with no tortuosity. The fourth row/second column shows coupling between axial position and RAO, which is not large. Here, TFP-99p appears to decrease monotonically with axial position, though with a slight rightward shift in the gradient at low and high RAO.

The third and fourth columns show remaining couplings, namely between lesion length and either SMB or RAO and between SBM and RAO (all at a maximum diameter of 4.0 cm). Generally, lesion length appears to couple nonlinearly with the other *g*_*i*_ (all fourth-row panels), though this coupling is weaker with SBM and RAO. Interestingly, longer or shorter lesions can be more thrombogenic while those of intermediate length can be less so. The saddle points within the length maps are non-intuitive, but aspect ratio and volume, which impact *ECAP*, play roles. Certain aspect ratios may be protective at higher diameters. Finally, the fourth row/fourth column shows the effect of Spinal Bending Magnitude (*g*_4_) on RAO, which is nonlinear but generally weak. The lower left region of the map contains lesions that have no tortuosity and a more distal left renal artery origin (and are close to being bilaterally symmetric). These lesions appear to be more thrombogenic, which may seem counter to the idea that both symmetry and distance from proximal branching vessels protect against thrombus formation.

Figure 5B shows similar maps, though focusing on vortical structures. The first column shows the coupling of diameter (abscissa) with axial position as well as three metrics of vortical structure (VS): VS_Bending_, VS_Area_, and VS_Depth_. Again, maximum diameter is a dominant morphological feature, though modulated by vortical structures. The first row/first column shows the coupling between diameter and axial position, which is similar to that in Fig. 5A except that the interpolations and mappings are within a different space, Ξ'. The first column/second row shows the coupling between maximum diameter and VS_Bending_. Recall that VS_Bending_ represents the deviation of the center of the vortical structure from the centerline of the vessel, which, due to the anterior deflection of SBM, is always posterior. Similar results are in the third and fourth rows/first column, except for VS_Area_ and VS_Depth_. Low values of these metrics of the vortical structures combined with large lesion diameters result in high values of TFP-99p. It seems, for example, that high VS_Bending_, that is, deflection of the VS from the centerline towards the walls, is mildly protective. Recalling that VS_Area_ represents the cross-sectional area of the VS orthogonal to the centerline at the point of break-up, VS_Area_ appears to be insignificant at low values of maximum diameter but important at high values. When VS_Area_ is large, the VS is close to the wall and decreases *ECAP*. Beyond a VS_Area_ of 1.0 cm^2^, the diameter of the lesion no longer appears to be relevant and the lesion is protected from thrombus. Finally, recall that VS_Depth_ refers to the distance the VS traverses within the lesion prior to break-up. Results (fourth row/first column) suggest that the VS passes through the lesion at high VS_Depth_, not breaking up and not releasing shear-activated platelets to regions of high *ECAP*, thus resulting in low *TFP*. Indeed, diameter becomes irrelevant at high VS_Depth_ and the gradient is intensified at low VS_Depth_.

The second column shows couplings between axial position and vortical structures. As it can be seen, lower values of the VS metrics tend to associate with a higher potential for thrombosis. When assessing VS_Area_ and VS_Depth_ in terms of axial position of the lesion, we also see local *TFP* maxima close to (but not coinciding with) the bottom left corner of the maps, thus more distal lesions are slightly more vulnerable to thrombosis than proximal lesions given similar values of the VS metrics. This finding may be because distal lesions tend to exhibit high *ECAP* regions, especially close to the iliac bifurcation. Activated platelets that are released proximally (at minimal VS_Depth_) or farthest from the aneurysmal wall (at minimal VS_Area_) may need to travel a relatively far distance to reach susceptible regions, thus resulting in lower *TFP*. Finally, the third and fourth columns show coupled effects among different metrics of the vortical structures. These also exhibit local maxima and saddle points, but changes are minimal despite the complex topography. Again, lower values of VS_Bending_, VS_Area_, and VS_Depth_ are more thrombogenic, consistent with other maps.

**Figure 6:**
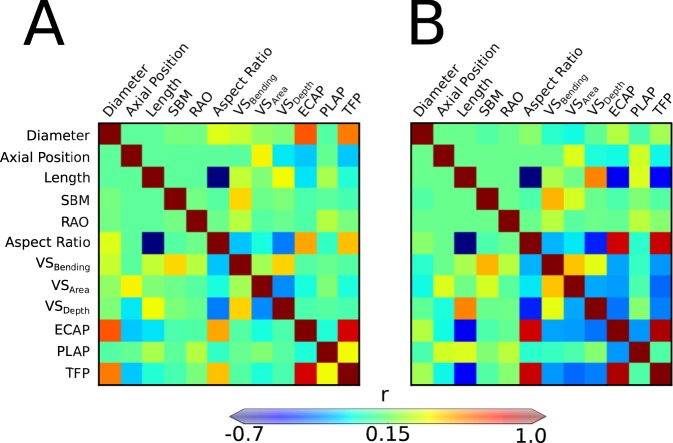
Symmetric correlation maps contrasting morphological (both direct and indirect) and hemodynamic metrics: maximum lesion diameter (Diameter), Axial Position, Axial Length, Spinal Bending Magnitude (SBM), renal artery offset (RAO), ratio between Diameter and Length (Aspect Ratio), three vortical structure metrics (VS_Bending_, VS_Area_, and VS_Depth_), *ECAP*, *PLAP*, and *TFP*. These maps were built considering all 179 simulations (left) versus only those simulations for AAAs with a maximum diameter greater than 4.5 cm (right). Note that *TFP* correlated strongly with maximum diameter but especially with *ECAP*, which suggests that *ECAP*could be a computationally less expensive surrogate.

Figure 6 provides another means of visualizing possible correlations amongst the different geometric and hemodynamic metrics, including the five key geometric parameters *g*_*i*_, lesion aspect ratio (a derived metric), the three VS metrics, and finally *ECAP*, *PLAP*, and *TFP*. Panel A shows results for all simulations. As it can be seen, these five geometric parameters were mutually independent (the largest correlation was between SBM and diameter, *r* = 0.055), as desired of potentially useful clinical metrics. Of these morphological features, consistent with findings in Figs 3–5, the maximum diameter of a lesion has the strongest direct correlation with *ECAP* (*r* = 0.66) and *TFP* (*r* = 0.59), followed by the aspect ratio (*r* = 0.50 for *ECAP*, *r* = 0.45 for *TFP*), which was determined largely by lesion length. Axial position (from the aorto-iliac bifurcation) correlated negatively with *ECAP* (*r* = −0.26) and *TFP* (*r* = −0.26), consistent with more proximal lesions tending to be less thrombogenic (Fig. 5), which may be due to the disturbed flow that occurs near branching vessels. For example, the entrance length was insufficient to reestablish laminar flow in the more proximal lesions and the coherent vortical structures seen in more distal lesions were disrupted, leading to greater mixing, higher *ECAP*, and lower *TFP*. Indeed, axial position correlated weakly with VS_Area_ (*r* = 0.32) and inversely with VS_Depth_ (*r* = −0.15), implying that vortical structures in proximal lesions tend to deflect from the centerline, widen towards the walls, and break-up early, all indicative of highly disturbed flows. VS_Area_ correlates weakly with VS_Bending_ (*r* = 0.16) and inversely so with VS_Depth_ (*r* = −0.33), consistent with the above. VS_Depth_ correlates with VS_Bending_ (*r* = 0.42), suggesting that VSs that penetrate deeper into a lesion tend to move off-center before breaking apart (often a direct consequence of SBM). Of note, *ECAP* correlated strongly with *TFP* (*r* = 0.86), while *PLAP* correlated less well (*r* = 0.33), suggesting that *ECAP*, which is less difficult to compute, may be an acceptable proxy for *TFP* if computational resources are limited.

Panel B in Fig. 6 shows similar results except for the larger lesions (*g*_1_ > 4.5 cm). Most correlations remain similar, though with a few notable exceptions. In large diameter lesions, *ECAP* correlates negatively with *PLAP* (*r* = −0.38), and the VS metrics correlate more inversely with *ECAP* (VS_Bending_: *r* = −0.40, VS_Area_: *r* = −0.38, VS_Depth_: *r* = −0.46) and *TFP* (VS_Bending_: *r* = −0.42, VS_Area_: *r* = −0.48, VS_Depth_:*r* = −0.43), suggesting that vortical structure dynamics are more important in large lesions. Aspect ratio also becomes a stronger determinant of *ECAP* (*r* = 0.86) and *TFP* (*r* = 0.87) in larger lesions.

### Parameter Sensitivity and Modeling Assessment

**Figure 7:**
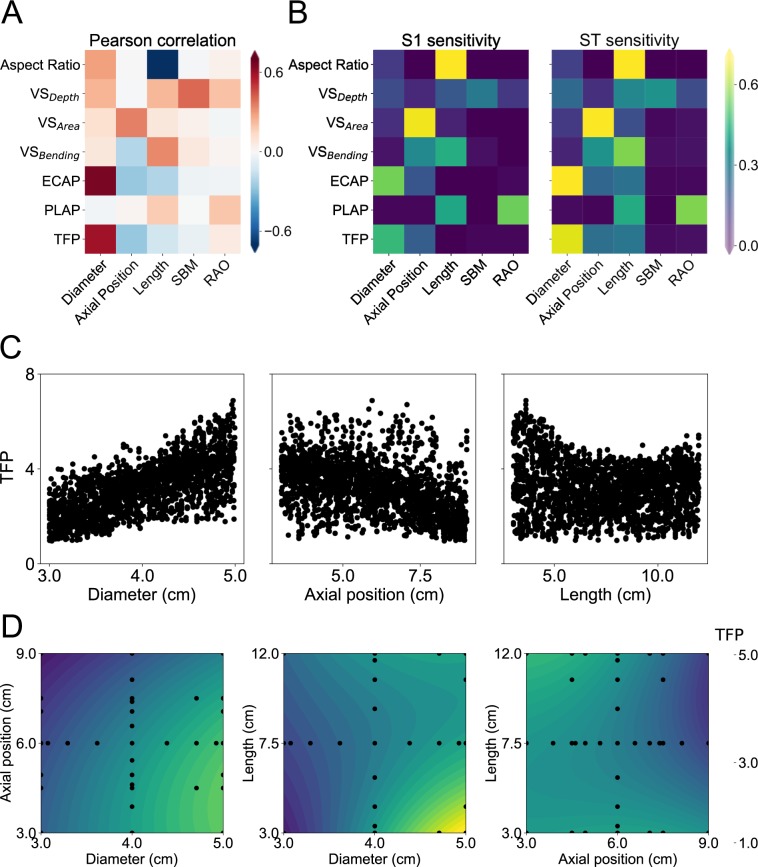
Comparison of different metrics of sensitivity. (**A**) Pearson correlation analysis, similar to the left box of Fig. 4. (**B**) Sensitivity indices computed via Sobol’s analysis. On the left, first order variance-based sensitivity indices (S1) reveal direct relationships between input geometric parameters (columns) and select outputs from the model. On the right, total effect sensitivity coefficients (ST) combine both direct and coupled contributions of the input parameters on select outputs. (**C**) Global Sensitivity analysis. Scatter plots indicate global behavior of *TFP* with respect to the 3 most influential parameters: diameter, axial position, and length of the lesion. Each solid dot represents a predicted value of *TFP* from the kriging surrogate model for combinations of input parameters sampled according to Saltelli’s scheme^55^. (**D**) High-dimensional model representation of *TFP* with respect to coupled variations of the 3 most influential parameters (two at a time).

A global variance-based analysis allowed us to quantify different objective measures of parameter sensitivity. Figure 7, panel A, shows Pearson correlation coefficients resulting from a linear regression analysis, while panel B depicts first-order and total effect sensitivity indices in the central and right heat-maps, respectively. While all sensitivity measures captured the dominant roles played by lesion diameter and axial position when varying model outputs, only the total effect indices highlighted contributions of lesion length, confirming that it acts primarily through coupled effects (e.g., aneurysms with high diameter/length ratio tend to be more thrombogenic). Panel C shows scatter plots of TFP values as a function of the three most influential geometric parameters (i.e., diameter, axial location, and lesion length) as predicted by the kriging surrogate at 12,000 locations of the hyperparameter space. The plots highlight the aforementioned trends of TFP increasing with lesion diameter and decreasing for more proximally located aneurysms; the more complex relationship between TFP and lesion length is also seen in the rightmost plot. The scatter in these plots highlights further the modulating effects of the multiple morphological features. Effects of varying parameters a couple at a time are shown in Panel D in terms of high-dimensional model reductions (HDMRs) of *TFP* variations. Unlike that shown in Fig. 6, generating HDMR contour plots did not require any explicit assumptions on the values of the remaining 3 input parameters that are not shown explicitly. Somewhat surprisingly, the plots resemble strongly the corresponding contour plots in Fig. 6, panel A.

**Figure 8:**
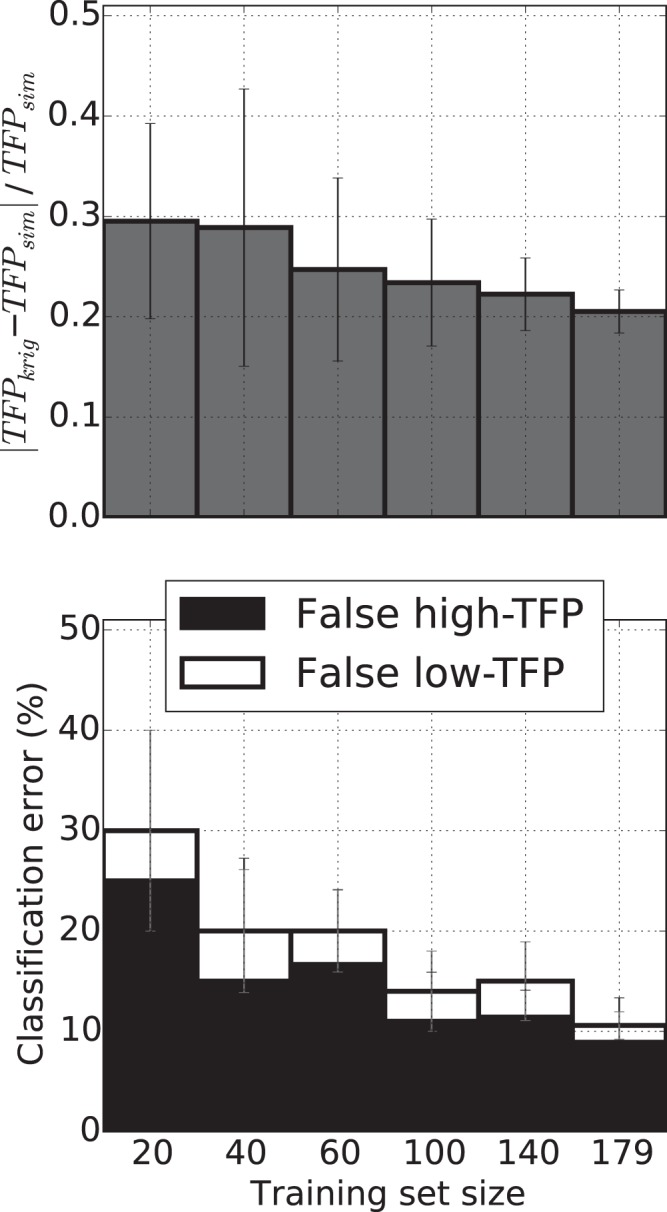
Assessment, via a 5-fold cross-validation of performance, of the kriging approximation as both a predictor of *TFP* and a classifier of high- or low- *TFP *lesions. Top panel shows the average relative discrepancy in *TFP* values between kriging predictions and actual simulations as a function of the training set size. Error bars represent variations observed when switching among the 5-fold splits of the training set. Bottom panel shows the percentage of lesions misclassified by the kriging approximation when using a *TFP* threshold of 2.5 to separate between high-*TFP* (black bars) and low-*TFP* (white bars) aneurysms. Meaning of the x-axis and error bars is same as for top panel.

Finally, to evaluate whether the number of full simulations was sufficient, we ran 5-fold cross-validation tests on training sets of varying sizes built with randomly selected entries from our 179 full simulations. The top panel in Fig. 8 shows average relative errors when approximating *TFP* via kriging rather than from full hemodynamic simulations. As expected, larger training pools led to better accuracy, with initially fast improvements that tended toward a plateau. Expanding the training set from 20 to 60 simulations yielded a ~5% decrease in average error (from ~30% to ~25%), while an improvement of only ~1.5% (from 22% to 20.5%) was observed when 39 simulations were added to a training pool with 140 entries. We also assessed performance of the kriging model as a classifier of high- versus low-*TFP* lesions separated by a *TFP* threshold value of 2.5. Such categorization subdivided the 179 simulations into a low-*TFP* group with 44 members and a high-*TFP* one with 135 members. The bottom panel shows the percentage of aneurysms misclassified by the kriging approximation for both of these categories, and for training pools of varying sizes. As expected, the largest training set yielded the fewest misclassifications, amounting to ~10.5% of the considered lesions. Most of those (~9% of the total) were falsely classified as high-*TFP* aneurysms, a finding that persisted even when increasing the *TFP* threshold from 2.5 to 3.0 (whereby 14% of the lesions were erroneously labeled as high-*TFP* out of 18% misclassified aneurysms). Predictions from the kriging model could therefore be considered conservative, with only a few lesions (i.e., only 1.5% or 4% of all aneurysms for the 2.5 or 3.0 thresholds, respectively) erroneously labeled at a low-risk of thrombogenesis.

## Discussion

There is now tremendous information regarding the histopathology, biomechanics, and clinical history of abdominal aortic aneurysms^3,8–10^, yet clinical decisions regarding intervention continue to rely primarily on the maximum dimension of the lesion or its rate of enlargement. Increasing evidence suggests, however, that ILT plays important roles in lesion enlargement and possible rupture^1–3^ and there is motivation to include predictions of thrombus formation within an overall clinical decision rubric. Notwithstanding many prior studies on the biosolid or biofluid mechanics of ILT in AAAs, both idealized and patient-specific^5–7,11–14^, the pronounced patient-to-patient variability in lesion geometry has delayed general understanding. In contrast, studying idealized lesions is advantageous for one can prescribe and assess carefully individual and coupled morphometric features and hemodynamic characteristics that might otherwise be masked by confounding variations within patient-specific geometries.

In particular, we reasoned that intra-aneurysmal hemodynamics, and thus thrombus formation, depends largely on coupled effects of multiple morphological features of the diseased abdominal aorta. As a first study of such effects, we selected five parameters that collectively account for considerable patient variability (cf. Fig. 1). Maximum lesion diameter is an obvious metric to consider, as is the overall length of the lesion (thus addressing the key non-dimensional parameter of length/diameter). Vessel curvature, or tortuosity, can have significant effects on the hemodynamics, including generation of secondary flows, and was deemed important to include. Similarly, proximal branch sites and angles can influence flows distally, with effects expected to be more dramatic in proximally versus distally located AAAs. Finally, of many possible considerations of proximal branch geometry, we focused on renal artery positioning since asymmetric renal arteries can break the symmetry of the flow into the lesion. Even with only five parameters, the curse of dimensionality leads to considerable computational challenges. Like patient-specific simulations^5^, calculating hemodynamics (involving unsteady 3-D Eulerian and particle-tracking Lagrangian analyses) within idealized lesions placed within a representative human abdominal aorta is computationally expensive, with each simulation requiring approximately 1000 CPU-hours on a high-performance cluster (128 CPU cores) and generating nearly 300 GB of raw data each. A straightforward parametric study was therefore impractical and so too a Monte Carlo approach was deemed not viable.

We thus sought to reduce the computational expense by minimizing the number of simulations that would be needed while enabling reliable interpolations across the 5-D parameter space. Adaptive sparse grid collocation was selected based on prior successes in studying geometric effects on hemodynamics^15^. As outlined in the Supplemental Information, multiple levels of selection led to full simulations at 179 collocation points, which in turn enabled reliable interpolation via kriging with a predicted low classification error (less than 4%) for false positives of low *TFP*. Not surprisingly, maximum diameter correlated well with high *TFP*, consistent with the clinical observation that larger lesions tend to harbor an ILT that often forms within the region of greatest aneurysmal bulging, namely ILT exists in up to 95% of lesions 3.5 cm or more in diameter^16^. Nevertheless, careful evaluations of select groups of simulations revealed couplings amongst multiple parameters (Figs 4 and 5), some not necessarily intuitive. For example, lesions with equal maximum diameters had very different thrombus formation potential depending on axial position, which was likely influenced dramatically by the integrity or break-up of the vortical structures^7^.

Importantly, interpolation-based maps from kriging suggested that, given the same diameter, increased thrombus formation potential may arise in lesions that are located more distally and/or are shorter; spinal curvature and renal artery offset affected the results much less. Because multiple morphometric features contribute to the hemodynamics proximal to (which influences platelet activation) and within (which influences endothelial vulnerability) the aneurysmal region, single metrics such as maximum diameter are unlikely to be predictive. Indeed, it has similarly been suggested that, despite its continued clinical usage, a simple metric such as maximum diameter is not as good of a predictor of aneurysmal rupture potential as local wall stress^17^, which can incorporate information on wall geometry, properties, and applied loads. Given that AAAs rupture when wall stress exceeds wall strength, multi-factorial metrics have been introduced to predict rupture potential based on wall strength^18,19^. Using multiple linear regression, such models can include geometric metrics such as normalized maximum diameter but also effects such as medical history and sex. Until we understand precise mechanisms, and perhaps even thereafter, there is similarly a need to investigate potential effects on ILT due to age, sex, past medical history, blood composition, prescription drugs, and potential co-morbidities such as hypertension or diabetes. Of course, consideration of such metrics will demand large patient-specific data sets, which was beyond the present scope. This study was clearly but a first assessment, with consideration of idealized lesions simplifying the parametric evaluation. We computed and compared peak *ECAP* and *TFP* by taking the 99^th^ percentile of luminal surface fields. Both metrics tended to be highest within the aneurysmal domain, rendering both ECAP-99p and TFP-99p potentially useful indices. Other metrics or reductions of field quantities could be envisioned. Moreover, other geometric parameters of potential importance could be considered. Among these, cross-sectional eccentricity (cf. Fig. 1), the degree and direction of bulging of the aneurysm from the natural curvature of the infrarenal aorta, the angle of the iliac bifurcation, and the presence of an extra renal artery (which manifests in approximately one third of all cases)^20^ could be considered. There is similarly a need to consider possible effects of peripheral vessel disease, such as stenosis or occlusion, and effects of spinal cord injury or lower limb amputation, all of which can adversely affect AAA hemodynamics^21–23^. Such simulations were simply beyond the present scope, particularly noting that one would need to appropriately adjust outlet conditions to account for remodeling of the contralateral iliac/femoral in cases of unilateral disease or amputation, hence increasing the parametric space considerably.

Increasingly sophisticated multiscale and/or systems-based models are becoming available for studying thrombus formation and deposition in complex hemodynamic fields^24–27^. Such models offer the promise of understanding mechanisms, which in turn could point to targeted therapeutics, and thus should be pursued in the future. Nevertheless, such studies require identification of many model parameters, and particle-based models remain computational impractical for large computational domains such as the human infrarenal aorta and distal common iliac arteries. Hence, we employed a simpler phenomenological model of thrombogenicity that has been validated against results from 9 patients^5,6^ and allows reasonable computational solutions (still ~1000 CPU hours each) to explore a 5-D geometric parameter space. We submit that, given that thin ILTs appear to incite a dramatic proteolytic insult to the aneurysmal wall^28–30^, having a computational model that depends primarily on easily obtained imaging based information and predicts well when (and where) an ILT will form^5^ could be sufficient to time clinical intervention during potential periods of rapid aneurysmal remodeling and expansion^4^. Mechanistic models, in turn, could help inform that clinical intervention and may not need to be patient-specific. Clearly, much remains to be accomplished before such models become part of the standard of care.

In conclusion, this paper presents the first melding of methods of computational hemodynamics, stochastic collocation, and Kriging to examine potentially coupled effects of common morphological features on ILT formation in AAAs. We have also proposed novel methods to extract and interpret quantitative hemodynamic features that drive ILT formation, revealing for example the importance of vortical structure dynamics in differently sized and shaped lesions. Simple maps (Fig. 6) suggest that *ECAP* correlates well with *TFP*, which is important from a practical perspective. *ECAP* can be determined via a standard (Eulerian) solution of the Navier-Stokes equations, not requiring the less straightforward (Lagrangian) particle tracking that is essential for calculating *TFP*. Hence, consistent with findings of thrombus formation in iliac artery aneurysms^31^, *ECAP* may be a computationally less expensive alternative for predicting possible thrombus formation. Regardless, computational studies are clearly critical for predicting thrombogenicity given the identified nonlinear couplings amongst parameters and they suggest that simple multilinear regressions will not be sufficient to determine a single scalar metric having clinical utility.

## Methods

### Morphological Quantification

Information from previous studies reported in the literature^32,33^ as well as from direct measurements on nine patient-specific reconstructions that we analyzed previously^5,6^ suggested reasonable ranges for each of the five parameters (Table 1) that are revealed by Fig. 1.

### Idealized geometric model

Unsteady, 3-D simulations of patient-specific hemodynamics within the abdominal aorta are computationally expensive, particularly when computing *TFP*, which requires standard Eulerian simulations via Navier-Stokes solutions plus Lagrangian particle tracking in post-processing to infer platelet strain histories. Spatial discretization of the former often requires 5+ million elements and temporal resolution often requires 3600+ time steps per cardiac cycle, with four or more cycles common to ensure convergence of the unsteady solution^6^. Total CPU time can often exceed 1000 hours per patient-specific model, which can be reduced in real time using parallel computing but is nonetheless expensive. Notwithstanding the difficulty of obtaining and reconstructing large numbers of patient-specific medical images to generate models of the hemodynamics, the number of geometries that can be investigated practically within a standard parametric study is limited by the computational expense. To facilitate a larger parametric study, we combined an analytical descriptor to define classes of AAA geometries and a statistical approach (sparse grid collocation to identify optimal parameter combinations and kriging to interpolate results) to reduce the total number of full simulations needed to characterize the five-dimensional parametric space (see below).

Inspired by previous work linking AAA morphology with the wall biomechanics^34^, each idealized aneurysm was modeled while assuming circular cross-sections orthogonal to a specified centerline, with luminal radius *r* given as a function of axial location *z* along that centerline:

1 $$r(z)={e}^{-{(\frac{{c}_{1}}{{r}_{IAA}})}^{2.42}{|\frac{z-{z}_{0}}{{l}_{o}}|}^{{c}_{2}}}[{r}_{AAA}-{r}_{IAA}-{c}_{3}(\frac{{(z-{z}_{0})}^{2}}{{r}_{IAA}})]+{r}_{IAA},$$

where *r*_*IAA*_ is the radius of the healthy segment of the infrarenal abdominal aorta (IAA), *r*_*AAA*_ is the maximum radius of the AAA (that is, *g*_1_/2), and *z*_0_ is the axial position of the maximal radius (cf., *g*_2_), all in mm. Together, the model parameters *c*_1_ (unit of length), *c*_2_ (unit-less when axial distance *z* is normalized per unit length, *l*_*o*_ = 1 mm), and *c*_3_ (unit-less) dictate the length (*g*_3_) and curvature (*g*_4_) of the aneurysmal dilatation. To approximate aneurysmal curvatures for multiple lesion lengths and diameters, we let *c*_1_ = 0.2 mm and *c*_3_ = 6.25⋅10^−4^, while *c*_2_ was varied according to a best-fit regression to match the desired length of the lesion (see below).

The centerline of the lesion was parameterized by the axial coordinate *z*, while the *x*-axis points to the left of the patient and the *y*-axis points anterior. The centerline is given by:

2 $$CL=[\begin{array}{c}x\\ y\\ z\end{array}]=[\begin{array}{c}\begin{array}{c}0\\ {c}_{4}erf({c}_{5}(z-{z}_{1}))\end{array}\\ z\end{array}],$$

where parameters *c*_4_ (unit of length) and *c*_5_ (unit of inverse length) specify, respectively, the desired forward bulging, or deflection, and angle of deflection, *θ*. The parameter *z*_1_ defines the bending offset where maximal bulging angle *θ* is achieved. We let *z*_1_ = 60 mm from the iliac bifurcation. The parameters *θ*, *c*_4_, and *c*_5_ are related via:

3 $$\theta =arctan(\frac{-2{c}_{4}{c}_{5}}{\sqrt{\pi }})\leftrightarrow {c}_{4}=\frac{-\sqrt{\pi }}{2{c}_{5}}tan(\theta ).$$

This relation provides two degrees of freedom in ascribing curvature with aortic dilatation. To limit this to one variable, *c*_5_ was set to $\frac{1}{6}\sqrt{\frac{\pi }{3}}$ mm^−1^, which yields a forward deflection (i.e., *c*_4_, *g*_4_) of 30 mm when *θ* = 30°. This selection allows the entire span of the anterior bulging to remain within the infrarenal aorta (i.e., prior to the iliac bifurcation) without any need to vary the curvature of the suprarenal aorta. Distal to the iliac bifurcation, centerlines for the iliac arteries follow a reflection of the above equation. The final centerline approximates the geometry observed *in vivo* due to the curvature of both the spine and the AAA itself.

It is important to distinguish the implicit parameters *c*_*i*_, which are used in the radial, centerline, and bending equations above, and the explicit parameters *g*_*i*_, which define the desired morphological features of each aneurysm. We used Mathematica 10.0 (Wolfram Research, Champaign IL) to perform a mapping between these two sets of parameters. With *c*_1_, *c*_3_, and *c*_5_ fixed, *c*_4_ = *g*_4_, and the desired spinal bending parameter *θ* determined via Equation (3), *c*_2_ was computed via a numerical interpolation for each specified radius and length. Specifically, roots of *r*(*z*)−1.1*r*_*IAA*_ were computed for different values of *c*_2_ and *r*_*AAA*_ and the Euclidean distance between these roots defined the length of the aneurysm (*g*_3_), recalling that the ends of the aneurysm are defined by a 10% increase in radius from *r*_*IAA*_. The resulting set of 37 tuples spanned maximum diameters *g*_1_ ∈ [30, 50] mm, lengths *g*_3_ ∈ [26.68, 159.88] mm, and values of *c*_2_ ∈ [1.72, 3.02]. Each value of *c*_2_ was then used with other values *c*_*i*_ to generate each lesion having the desired *g*_*i*_.

In addition to the idealized lesion, the overall computational domain included the iliac arteries, renal arteries, superior mesenteric artery (SMA), and celiac artery (Fig. 1) since each of these branches and associated flows can affect flow within an AAA. In particular, the axial offset between the ostia of the two renal arteries (*g*_5_) was included for it can break the symmetry of the flow into the lesion and thus affect vortical structures. To minimize entrance effects, the computational domain was extended from the suprarenal abdominal aorta (SAA) into the distal descending thoracic aorta using segments of appropriate length and diameter (Fig. 1). The inferior mesenteric artery, which typically stems from the anterior portion of the IAA, was omitted because it is rarely identifiable in AAA scans. The lengths, diameters, branching origins, and branching angles of all included vessels are listed in Table 2. Lengths are computed along the centerline, with axial position representing either the ostia of a branching vessel or the axial domain of an aortic segment. All axial positions are given via the *z* coordinate, which is zero at the iliac bifurcation. The branch angle represents the absolute value of angular deflection from the abdominal aorta. Clock angles represent the direction of branching of a given vessel.

**Table 2:** Dimensions and Geometry Defining the Computational Domain.

Artery	Diameter (cm)	Axial Position (cm)	Length (cm)	Branch Angle (degrees)	Clock Angle (degrees)
Thoracic Aorta	2.20	17.0–27.0	10.0	N/A	N/A
Celiac	0.80	17.0	5.0	70.0°	0.0°
Superior Mesenteric	0.80	14.5	8.0	40.0°	0.0°
Left Renal	0.70	12.0 + RAO	7.0	60.0°	90.0°
Right Renal	0.70	12.0	7.0	60.0°	270.0°
Infrarenal Aorta	1.80 (baseline)	0.0–12.0	12.0+*	N/A	N/A
Left Iliac	1.00	0.0	10.0+*	25.0°	90.0°
Right Iliac	1.00	0.0	10.0+*	25.0°	270.0°

*Depends on spinal bending magnitude. Equal to displayed values when θ = 0.

### Discretized geometric model

Model generation largely follows prior methods^5,6^, with some notable differences. A custom Python code based on the open-source Visual Toolkit (VTK) library (Kitware Inc., Clifton Park, NY) generated circular cross-sections at regular intervals for each idealized vessel, centered on and orthogonal to the centerline. These cross-sections were imported into a custom version of *SimVascular*^35^, an open-source tool used to generate vascular models and simulate blood flow^36^. Segmentations were lofted with Non-Uniform Rational Basis Splines (NURBS) to generate a 3-dimensional surface model, and a blending procedure smoothed transitions at vessel branch points to minimize simulation artifacts. A tetrahedral mesh was generated for each geometry using MeshSim libraries (Simmetrix Inc., Clifton Park NY) to form a finite element model with a nominal tetrahedral element size of 1.0 mm, with mesh refinement near the luminal surface (Fig. S1 in the supplemental material). Algorithms available in the open-source Vascular Modeling Toolkit (VMTK)^37,38^ marked the domain with features helpful for post-processing, such as generating centerlines and splitting the external surface into segments of the various vessels. Models included, on average, 2.4 ± 0.2 million elements.

### Statistical learning of the parameter space

Our primary goal was to investigate parametrically the influence of vascular and aneurysmal morphologies on infrarenal hemodynamics and to predict potential ILT formation. Because of expected complex nonlinear coupling of such morphological features (e.g., lesion position and curvature should modulate the effects of maximal diameter), we sought to examine myriad combinations of five key parameters on the intra-aneurysmal *TFP*. Yet, appropriate spanning of this five-dimensional space over pathophysiological ranges (Fig. 2A, Table 1) precluded a standard parametric sensitivity study. For example, simply considering all combinations of five different parameters, even with only five different values, would require 5^5^ = 3125 full simulations. One approach to reduce the burden of spanning high-dimensional parameter spaces is the Chebyshev sparse grid stochastic collocation method^39–41^. Let Ξ represent the stochastic geometric parameter space, with dimensions defined by *g*_*i*_(*i* = 1, 2, …, 5). Rules provided by the Chebyshev sparse grid method yielded points *ξ*_*l*,*j*_(*l* = 0 ... 3, *j* = 1... *n*_*l*_) that would efficiently probe Ξ. As is common in Smolyak-type constructions^15^, our sparse grid was built hierarchically, meaning that the set of collocation nodes was progressively expanded to reach the desired accuracy. We constructed 4 levels (*l* = 0 ... 3) of progressive refinement (see Supplemental Information), which resulted in collocation points at the roots of 5-D Chebyshev polynomials of degree 1 to 4.

After running simulations at collocation points for each refinement level, multiple hemodynamic metrics were extracted in post-processing, including wall shear stress indices, thrombus formation potential, and characteristics of vortical structures (see Sections 2.5–2.7). A 5-D Gaussian process regression (kriging) model with a radial basis function kernel was then trained to interpolate each of the hemodynamic features over Ξ. Our implementation used the open-source package GPy^42^. From this, the value and uncertainty of each metric was estimated at any point in Ξ, and thus for any complex geometry within the parameter space.

To minimize further the number of simulations needed, we employed the adaptive refinement strategy introduced by Sankaran and Marsden^15^. This allowed us to discard 62 out of 180 collocation points initially prescribed for refinement level 3, since results from levels 0, 1, and 2 showed that certain areas of the parameter spaces were already characterized sufficiently. Thus, we considered a total of 179 lesion morphologies. Recall that Fig. 2B shows a 3-D projection of Ξ to visualize collocation points while compressing the *g*_4_ and *g*_5_ dimensions. The nodal distribution reveals that Chebyshev grids tend to be denser along the mid-range and around boundary vertices of the parameter space. Also, asymmetries in the collocation points resulted from the adaptive refinement scheme, which revealed, for example, that limited new information is obtained when varying aneurysm length when at the smallest lesion diameter. Figure 2C details two slices of the 3-D projection along *g*_1_ − *g*_2_ and *g*_1_ − *g*_3_ mid-planes.

### Computational hemodynamics simulations

Full 3-D finite element solutions of the Navier-Stokes equations were computed for each of the 179 model geometries. Inlet and outlet boundary conditions were prescribed for rest conditions, which yields worst case scenarios for ILT since exercise may limit thrombus formation^5^. The volumetric flow rate over a cardiac cycle at the inlet of the descending thoracic aorta was obtained from Les *et al*.^43^ and the associated inlet velocity profile was computed as a Womersley pulsatile laminar flow^44^, with 15 Fourier series terms. The cardiac cycle was assumed to have a period of 0.9 seconds, corresponding to a heart rate of ~67 beats per minute. Outlet boundary conditions were specified using a lumped-parameter circuit model (Windkessel resistance-capacitance-resistance boundary conditions, see Fig. S1 in supplemental material), as is now common^45,46^. Values of normal outlet conditions were prescribed from Xiao *et al*.^47^. The blood was modeled as a Newtonian fluid, with a constant viscosity and density of 0.004 g/mm∙s and 0.0016 g/mm^3^, respectively. All vessel walls were assumed rigid similar to our previous studies wherein potential effects of this assumption were discussed and accepted^5^.

The incompressible Navier-Stokes equations were solved in *SimVascular*, using a stabilized finite element flow solver, to generate unsteady pressure and velocity fields within the 179 model aneurysms as well as in the normal aortic segments just proximal and distal to each lesion. The cardiac cycle was subdivided into steps of 0.001 seconds, with computational results saved every 5 steps (0.005 seconds). Simulations were performed for four cardiac cycles having a uniform period *T* = 0.9 seconds. To allow transient flow patterns to dissipate and to establish periodicity, we used results from the last cardiac cycle for post-processing and Lagrangian particle tracking simulations as described previously^5^.

Post-processing allowed computation of several biomechanical metrics from the velocity fields. Of primary relevance were two luminal surface metrics: a Time-Averaged Wall Shear Stress magnitude $(\overline{TAWSS})$ that is normalized by the mean value of *TAWSS* in the descending thoracic aorta, and a Oscillatory Shear Index (*OSI*) that measures changes in the *WSS* field due to complex oscillatory flows. The quotient of these metrics defines the Endothelial Cell Activation Potential (*ECAP*), which delineates regions of high *OSI* and low *TAWSS* where the vessel is thought to be particularly susceptible to thrombus formation^5,48^. *TAWSS*, *OSI*, and *ECAP* are given by the following point-wise metrics defined on the luminal surface:

4 $$TAWSS=\frac{1}{T}{\int }_{0}^{T}|{\tau }_{w}|dt\,,\,OSI=\frac{1}{2}(1-\frac{|{\int }_{0}^{T}{\tau }_{w}dt|}{{\int }_{0}^{T}|{\tau }_{w}|dt})\,,\,ECAP=\frac{OSI}{\bar{TAWSS}}$$

Using a normalized point-wise $\overline{TAWSS}$ allows uniform comparisons across different geometries.

### Vortical structure analysis

Shear stress indices such as *TAWSS*, *OSI*, and *ECAP* are highly affected by the dynamics of vortical structures near the wall. To investigate how morphological features of a lesion might influence the path taken by vortical structures throughout the cardiac cycle, we examined *λ*_2_ iso-surfaces^7^. *λ*_2_ is the second largest eigenvalue of the tensor ***D***^2^ + ***Ω***^2^, where ***D*** = 0.5(***l*** + ***l***^*T*^) is the symmetric part of the spatial velocity gradient tensor ***l*** and ***Ω*** = 0.5(***l*** − ***l***^*T*^) is its skew-symmetric counterpart. A surface of a negative *λ*_2_ encloses a pressure maximum, which typically indicates the presence of a vortex^49^. To analyze paths and other key features of vortical structures during a cardiac cycle, we built iso-surfaces directly on minimum values of *λ*_2_ at each location. As such, our plots merged the dynamic evolution of the vortical structure into a single snapshot showing envelopes of the traces of the vortices. While this representation tends to hide temporal features of vortex dynamics (e.g., it does not discriminate whether a vortical structure moves on quickly or stations longer within a given region), it simplifies the analysis of vortical paths and their locations. Specifically, we measured for each case: (1) *VS*_*Depth*_, namely how deep the main vortical structures penetrated into the aneurysm before breaking into smaller structures; (2) *VS*_*Area*_ (in mm^2^), the cross-sectional area enclosing vortical structures when they break-up; and (3) *VS*_*Bending*_ (in mm), the distance between the centerline of the aneurysm and the center of the cross-sectional area where the vortex breaks-up.

#### Thrombus formation potential

Standard hemodynamic simulations allow one to compute *ECAP*, a measure of wall susceptibility to thrombus deposition. A second significant contributor to thrombogenesis is the presentation, then aggregation, of activated platelets at the susceptible site. This activation can depend highly on the mechanical shear history^7,50–53^. To determine which regions of the luminal surface were presented with shear-activated platelets, we used Lagrangian particle tracking simulations, as described previously^5^. Briefly, a secondary mesh with nominal resolution of 0.5 mm and increased curvature refinement was generated to fill the computational domain and a particle was placed at each node of the mesh. We then advected the platelets backward through time, looping over 8 cardiac cycles using flow results to track the trajectories and shear history of model platelets that ended up adjacent to the vascular wall. Ten simulations were run in parallel, each with a different evenly-distributed injection time in the cardiac cycle, and results were averaged. The particle tracking simulations were used to calculate the PLatelet Activation Potential (*PLAP*), proposed by Shadden and Hendabadi^53^ and employed by Di Achille *et al*.^5^. *PLAP* is a non-dimensional volumetric index of the cumulative shear rate history experienced by particles that arrive at a particular point in the domain (i.e., the starting point prior to advection backward through time). It is defined as

5 $$PLAP({\boldsymbol{x}},t)={\int }_{t-8T}^{t}|{\boldsymbol{D}}({\boldsymbol{x}}(\tau ),\tau )|\,d\tau ,$$

where |***D***(*x*(*τ*),*τ*)| is the Frobenius norm of the symmetric part of the spatial gradient of the velocity tensor, *t* is the time of injection of the particle, and *T* is the period of the cardiac cycle. A surface *PLAP* is generated by averaging the volumetric *PLAP* as described in Di Achille *et al*.^5^ and normalizing by its average value in the distal descending thoracic aorta. *PLAP* provides significant insight into stagnant flows, particle attractors, and the typical delivery distribution of activated platelets to the endothelium, all of which are relevant to thrombogenesis.

The *ECAP* and *PLAP* were combined to generate a Thrombus Formation Potential (*TFP*):

6 $$TFP\equiv ECAP\cdot PLAP=\frac{OSI\cdot PLAP}{\overline{TAWSS}}.$$

Co-localization of wall priming (high *ECAP*) and advection of shear-activated platelets (*PLAP*) thus creates a surface index that predicts thrombus initiation at a given location and time^5,6^. This metric can be used as a proxy for thrombogenicity in a given lesion and thus has clinical applicability.

#### Global sensitivity analysis

Sobol’s variance-based sensitivity analysis provides an effective strategy to decompose the variance of output metrics in terms of relative direct and indirect contributions of the input parameters^54^. This analysis nevertheless requires extensive probing of the hyperparameter space to rule out potentially spurious correlations and to quantify objective sensitivity measures. To render the global analysis feasible in our case, we applied Sobol’s analysis on predictions from optimized kriging interpolations (see Section 4.4). Samples were collected following Saltelli’s scheme^55^, which in its most robust form requires *r*(2*k* + 2) kriging evaluations, where *k* is the number of input parameters and *r* indicates the resolution chosen to characterize each input dimension. Following common practice, we chose *r* = 1000 for a total of 12000 kriging evaluations. Sample selection and sensitivity indices were calculated using the open-source python library SAlib^56^. In particular, we computed and compared first-order and total effect sensitivity indices. We also used analysis of variance theory to generate alternative visualizations of the combined effects of morphological parameters on *TFP*. Specifically, we built high-dimensional model representations of *TFP* as functions of selected couples of inputs according to^57–60^

7 $$TFP({g}_{i},{g}_{j})=E(TFP|{g}_{i},{g}_{j}),\,i,j=1\ldots 5\,$$

where *g*_*i*_, *g*_*j*_ represent values of a selected input features, *E*(.) is the expected value operator and | indicates a conditional probability. The conditional expected values of *TFP* were computed via inference on 2-D Gaussian process regressions optimized on the 12000 extracted samples.

## Electronic supplementary material

Supplemental Information

## Data Availability

All input data needed to reproduce the results are provided in the text or tables.
