# Supplementary material for: Strongly Coupled Morphological Features of Aortic Aneurysms Drive Intraluminal Thrombus
Source: Sci Rep. 2018 Sep 5;8:13273. doi: 10.1038/s41598-018-31637-6 (PMC6125404; doi:10.1038/s41598-018-31637-6)
Supplement: Supplementary file 1 — Supplemental Information [file 41598_2018_31637_MOESM1_ESM.pdf]

Supplemental Information

Strongly Coupled Morphological Features of Aortic Aneurysms Drive Intraluminal Thrombus

D. Bhagavan[1,*], P. Di Achille[1,*], J.D. Humphrey[1,2]

[1]Department of Biomedical Engineering

Yale University, New Haven, CT, USA

[2]Vascular Biology and Therapeutics Program

Yale School of Medicine, New Haven, CT, USA

[*]These authors contributed equally.

Address for Correspondence:

J.D. Humphrey, Ph.D.
Department of Biomedical Engineering
Yale University
New Haven, CT 06520, USA
jay.humphrey@yale.edu
+1-203-432-6428

Additional Methods

Although patient-specific clinical assessments will ultimately be needed to confirm computational predictions of the myriad coupled factors that drive AAA thrombogenicity, the inherent patient-to-patient variability in lesion geometry renders it nearly impossible to glean understanding even from large numbers of patient-specific simulations. Indeed, despite considerable attention to the relationship between hemodynamics and thrombus formation, the number of available patient-specific simulations remains modest, as, for example, one patient in Basciano et al.[13], two patients in Biasetti et al.[7], three patients in O'Rourke et al.[58], six patients in Di Achille et al.[6], ten patients in Arzani et al.[59], and fourteen patients in Zambrano et al.[16]. Idealized models thus offer tremendous advantages in examining the contributions of diverse morphological features, as revealed by the important experimental study of Salsac et al.[60]. Nevertheless, experimental studies (requiring the fabrication of many model lesions) are also cost- and time-prohibitive when seeking to examine many different cases. Simulations based on select idealized lesions based on adaptive sparse grid collocation are thus a prudent alternative.

Sparse Grid Collocation

The spinterp toolbox[39] was used in MATLAB **(**Mathworks Inc., Natick MA**)** to generate points ξ_j for levels 0, 1, 2, and 3 of the sparse grid. Level 0 (L0) consists of one point, the standard geometry at the geometric center of the hyper-cubical parameter space (Figure 2). L1 consists of 10 additional points, extremes in one parameter and mid-ranges in the others. L2 consists of 50 additional points, extremes in two parameters and mid-ranges in the others as well as points that are intermediate in one dimension. L3 continues this trend, with a suggested 180 additional points. In principle, as additional iterations are completed, the predicted error diminishes and the algorithm converges. When all simulations fall below predetermined error tolerances, the algorithm terminates.

Adaptive Sparse Grid

Despite implementing a sparse grid collocation, the computational expense of individual simulations curtailed the number that could be run practically. Indeed, although full convergence of the grid did not appear to be achievable through L3, we sought to reduce the required number of simulations further. Sparse grid collocation operates independent of the behavior of the field it attempts to approximate. Although the field of interest is often nonhomogeneous and anisotropic, it can vary smoothly and minimally in certain regions of Ξ, while in other regions it may change rapidly. In the former regions, interpolation error is likely to be low, requiring fewer points to interpolate accurately. In

the latter regions, a higher density of simulations may be required to capture the behavior of the field. The classical stochastic sparse grid collocation algorithm, while guaranteed to converge, does not account for these possible characteristics, thus a large number of iterations may be required to converge. This characteristic results in a suboptimal number of simulations required to reach convergence for a specific metric.

Sankaran and Marsden[15] proposed an adaptive sparse grid algorithm that accounts for heterogeneity and anisotropy in the field of interest, refining the points needed for interpolation based on results of the previous iteration. This method can reduce the total computational expense significantly. We adapted their method to the present case (to reduce the required 241 simulations for L0-L3), making further refinements to account for the range of interest of our primary field, the Thrombus Formation Potential (TFP). Our primary interest was in lesions that are thrombogenic, defined by a 99th percentile TFP above a threshold of 2.5 to 3.0. To that end, we refined the adaptive sparse grid algorithm to prioritize those regions of Ξ when refining the grid.

The adaptive sparse grid algorithm was implemented as follows. Let d be the interpolation depth and n the number of dimensions. Let $f(g_1, \ldots, g_n) \equiv f(\bar{\xi})$ be the one-dimensional field of interest (or a component of a higher-dimensional field), where g_i are the geometric parameters and $\bar{\xi} \in \Xi$ is a point in the stochastic space. Let $\bar{c}_k \in \Xi$ be a collocation (grid) point, and $\mathrm{C_d}$ the cumulative set of standard sparse grid collocation points up to iteration depth d, with $\mathrm{C_d} \supset C_{d-1} \supset \cdots \supset C_0$. Let C_d^A be the cumulative set of adaptive sparse grid points generated by the algorithm at a given iteration depth. Then, let $\bar{c}_k^d$ be the set of $\bar{c}_k \in C_d \backslash C_{d-1}$, the set of new grid points belonging exclusively to a particular iteration d. We then designate two mutually exclusive categories of points. Let A be the set of active points and F be the set of fixed points, such that $A \cap F = \emptyset$ and $A \cup F = C_d$ for each interpolation. Next, for each $\bar{c}_k^d$, let N_k be the set of its Nearest Linear Grid Neighbors (NLGNs), defined as the collection of closest points $h_i \in \Xi$ within $1/d$ of $\bar{c}_k^d$ along each coordinate direction $\hat{g}_i$. Each $\bar{c}_k^d$ will have up to $2n$ NLGNs, one in each direction in each dimension. Next, let $f_d^I(\xi)$ be the value of $f(\xi)$ predicted by the interpolation generated after iteration d. From this, we can define an error function at each new adaptive grid point $\bar{c}_k^d \in C_d^A \backslash C_{d-1}^A$ after executing iteration d:

$$\varepsilon_d(\bar{c}_k^d) \equiv \frac{1}{2}\left[\left|\frac{f(\bar{c}_k^d) - f_{d-1}^I(\bar{c}_k^d)}{f(\bar{c}_k^d)}\right| + \left|\frac{f(\bar{c}_k^d) - f_{d-1}^I(\bar{c}_k^d)}{f_{d-1}^I(\bar{c}_k^d)}\right| \right]$$

This non-dimensional error function compares the predicted proportional difference in error between the predicted field value $f_{d-1}^{I}(\bar{c}_k^d)$ from the previous iteration and the true value $f(\bar{c}_k^d)$, obtained after running the simulation at $\bar{c}_k^d$ in the current iteration. The function also averages two expressions that normalize by the true and predicted values, respectively. This is done to mitigate the error magnitude when one or both values is close to zero. Finally, let τ be the predefined error tolerance and η be the predicted field threshold. The algorithm is as follows:

```
Run simulations at d=0 and d=1
Let d=1
$F = \emptyset$
while $\exists \bar{c}_k \notin F$ :
    d++
    for $\forall \bar{c}_k^d \in C_d^A \backslash C_{d-1}^A$ :
        if $\forall h_i \in N_k, \nexists h_i \in A$ :
            $\bar{c}_k^d \in F$
        else:
            Run simulation at $\bar{c}_k^d$
            if $\varepsilon_d(\bar{c}_k^d) < \tau$ or $f(\bar{c}_k^d) < \eta$ :
                $\bar{c}_k^d \in F$
Terminate
```

The first two iterations of the sparse grid, L0 and L1, must be run to create an initial interpolant. Initially, all points in an iteration are classified as active, implying the third iteration, L2, must be run in full. When all active points in an iteration are run, the algorithm compares the true value of the functional $f(\bar{c}_k^d)$ at each point to the predicted value of the interpolant from the previous iteration, $f_{d-1}^{I}(\bar{c}_k^d)$. If the error function falls within tolerance τ or if the field value $f(\bar{c}_k^d)$ is below the threshold η, the grid point $\bar{c}_k^d$ is designated as frozen; else, it remains active. After completing this process with all

points $\bar{c}_k^d$, the set of points $\bar{c}_k^{d+1}$ are generated for the next iteration. The iteration proceeds in a similar manner, with the following important criterion. The NLGNs of each point in the new iteration are computed. If all neighbor points $h_i \in N_k$, themselves collocation points of previous iterations, are frozen, then the corresponding point $\bar{c}_k^d$ is designated as frozen; else, it is designated active. Essentially, a point whose neighbors are all frozen (i.e. their interpolated field values fall within the error tolerance) is likely to have an interpolation that falls within the tolerance as well, and therefore does not need to be computed. Simulations are only run at active points, enabling a reduction in computational expense. Freezing points whose functional value is below threshold η does reduce the number of simulations required while increasing the interpolation error in regions where $f(\xi)$ is low; however, those regions are of less clinical interest, as they are not thrombogenic. When all collocation points in an iteration are designated as frozen, without the need to run simulations and compute $f(\bar{c}_k^d)$, the algorithm terminates.

The adaptive sparse grid algorithm was implemented in MATLAB. We used the one-dimensional field TFP-99p, the 99th percentile value of the TFP in a given lesion, as our functional $f(\xi)$. A threshold value of $\eta = 3.0$ and an error tolerance of $\tau = 0.10$ were used to refine the adaptive algorithm. An initial cohort of 61 high-fidelity geometries (L0, L1, and L2) were generated, and simulations completed. The adaptive algorithm suggested an additional 118 simulations (L3), which were also run. This resulted in 179 high-fidelity (full) simulations, a 94.3% reduction in computational expense from a simple grid with a resolution of 5 points per dimension (3125 points), and a 25.7% reduction in expense from the classical sparse grid algorithm, which would have required 241 simulations at the same iteration depth.

See manuscript for references

Figure S1

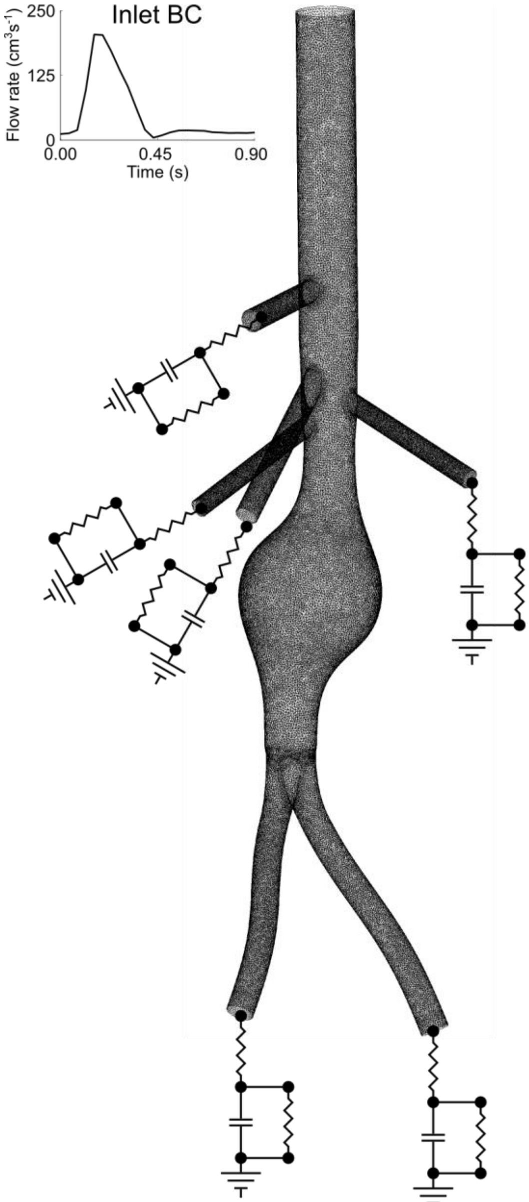

Figure S1. Domain discretization and boundary conditions for a representative lesion geometry. Suprarenal inlet flow-rate from Les et al.[43] was imposed as a Dirichlet BC under the assumption of a Womersley velocity profile. The computational domain had 6 outlets representing the celiac trunk, the SMA, the renal arteries, and the iliacs. All of them were modeled as 3-element Windkessel models weakly coupled as Neumann BCs on traction. Values for the electric analog components were selected from Xiao et al.[47]. See manuscript for references.

Table S1. Pearson correlations among independent geometric parameters, measures of vortical structures (VS), and thrombogenic indices (*ECAP*, *PLAP*, and *TFP*). Position is axial position measured from the iliac bifurcation. SBM – Spinal Bending Magnitude, RAO – renal artery offset, *ECAP* – endothelial cell activation potential, *PLAP* – platelet activation potential, and *TFP* – thrombus formation potential.

	Diameter	Position	Length	SBM	RAO	Aspect Ratio	$VS_{Bending}$	VS_{Area}	VS_{Depth}	ECAP	PLAP	TFP
Diameter	1.00	0.00	0.00	0.06	0.05	0.29	0.24	0.11	0.08	0.66	-0.02	0.59
Axial	0.00	1.00	0.00	0.00	0.00	0.00	-0.01	0.35	-0.20	-0.26	0.02	-0.26
Length	0.00	0.00	1.00	0.00	0.00	-0.86	0.24	0.08	0.34	-0.21	0.18	-0.13
Bending	0.06	0.00	0.00	1.00	0.05	-0.02	0.40	0.04	0.07	-0.04	0.00	-0.05
RAO	0.05	0.00	0.00	0.05	1.00	0.04	0.20	-0.02	0.02	-0.03	0.19	0.06
Aspect Ratio	0.29	0.00	-0.86	-0.02	0.04	1.00	-0.27	-0.15	-0.40	0.50	-0.10	0.45
$VS_{Bending}$	0.24	-0.01	0.24	0.40	0.20	-0.27	1.00	0.16	0.42	-0.02	0.03	-0.02
VS_{Area}	0.11	0.35	0.08	0.04	-0.02	-0.15	0.16	1.00	-0.37	-0.16	0.08	-0.15
VS_{Depth}	0.08	-0.20	0.34	0.07	0.02	-0.40	0.42	-0.37	1.00	-0.04	-0.02	-0.05
ECAP	0.66	-0.26	-0.21	-0.04	-0.03	0.50	-0.02	-0.16	-0.04	1.00	-0.14	0.86
PLAP	-0.02	0.02	0.18	0.00	0.19	-0.10	0.03	0.08	-0.02	-0.14	1.00	0.33
TFP	0.59	-0.26	-0.13	-0.05	0.06	0.45	-0.02	-0.15	-0.05	0.86	0.33	1.00

Table S2. Independent geometric parameters, measures of Vortical Structure (VS), and thrombogenic indices (*ECAP*, *PLAP*, *TFP*) for all 179 simulations selected to probe the parameter space. Position is axial position measured from the iliac bifurcation. SBM – Spinal Bending Magnitude, RAO – renal artery offset, *ECAP* – endothelial cell activation potential, *PLAP* – platelet activation potential, and *TFP* – thrombus formation potential.

AAA	Diameter (cm)	Position (cm)	Length (cm)	SBM (cm)	RAO	Aspect Ratio	$VS_{Bending}$ (mm)	VS_{Area} (mm^2)	VS_{Depth}	ECAP	PLAP	TFP
0	4	6	7.5	2.5	-0.50	0.53	3.9	92.1	0.40	4.56	0.78	3.03
1	3	6	7.5	2.5	0.75	0.40	8.6	50.3	0.66	1.95	1.10	1.92
2	4	3	7.5	2.5	0.75	0.53	9.0	72.4	0.70	3.37	0.93	2.63
3	4	6	12	2.5	0.75	0.33	3.0	97.9	0.42	5.30	0.77	3.41
4	4	6	3	2.5	0.75	1.33	2.8	111.7	0.55	5.00	0.95	3.98
5	4	6	7.5	0	0.75	0.53	3.9	74.3	0.67	5.31	0.82	3.78
6	4	6	7.5	2.5	2.00	0.53	8.8	95.9	0.48	3.91	1.01	3.03
7	4	6	7.5	2.5	0.75	0.53	10.8	75.7	0.59	2.94	0.98	2.48
8	4	6	7.5	5	0.75	0.53	9.4	81.5	0.52	3.41	0.99	2.80
9	4	9	7.5	2.5	0.75	0.53	2.7	116.5	0.37	3.43	1.03	2.94
10	5	6	7.5	2.5	0.75	0.67	16.5	82.7	0.68	3.69	0.91	3.02
11	3.2929	6	7.5	2.5	0.75	0.44	7.0	70.8	0.55	3.31	0.91	2.71
12	3	3	7.5	2.5	0.75	0.40	6.1	9.6	0.79	2.95	0.96	2.62
13	3	6	12	2.5	0.75	0.25	1.6	74.4	0.32	2.49	0.90	1.78
14	3	6	3	2.5	0.75	1.00	2.0	52.8	0.43	1.22	0.73	0.78
15	3	6	7.5	0	0.75	0.40	3.1	64.8	0.47	2.52	0.91	1.98
16	3	6	7.5	2.5	-0.50	0.40	1.7	73.0	0.58	3.01	0.90	2.25
17	3	6	7.5	2.5	2.00	0.40	3.6	66.6	0.49	2.34	1.03	2.22
18	3	6	7.5	5	0.75	0.40	7.6	75.1	0.57	2.47	0.84	1.84
19	3	9	7.5	2.5	0.75	0.40	1.4	86.9	0.42	1.43	0.58	0.71
20	4	6	3	5	0.75	1.33	2.5	50.2	0.39	4.41	0.81	3.24
21	5	3	7.5	2.5	0.75	0.67	4.9	78.2	0.60	6.25	0.92	4.76
22	4.7071	6	7.5	2.5	0.75	0.63	13.8	87.9	0.64	5.75	0.72	3.45
23	4	3	12	2.5	0.75	0.33	-0.1	1.9	0.87	4.89	0.98	4.33
24	4	3	3	2.5	0.75	1.33	1.3	51.7	0.47	4.79	1.04	3.94
25	4	3	7.5	0	0.75	0.53	3.9	68.8	0.31	5.44	0.88	4.33
26	4	3	7.5	2.5	-0.50	0.53	2.3	70.0	0.59	4.99	0.97	4.12
27	4	3	7.5	2.5	2.00	0.53	10.6	70.8	0.66	4.37	0.96	3.24

28	4	3	7.5	5	0.75	0.53	4.2	53.6	0.32	4.32	0.98	3.71
29	4	3.8787	7.5	2.5	0.75	0.53	2.7	64.8	0.45	4.89	0.95	3.97
30	4	6	10.68195	2.5	0.75	0.37	8.4	15.9	0.81	5.07	0.83	3.44
31	4	6	12	0	0.75	0.33	6.7	93.9	0.48	3.99	1.02	3.22
32	4	6	12	2.5	-0.50	0.33	3.9	87.1	0.29	4.38	0.98	3.77
33	4	6	12	2.5	2.00	0.33	7.7	93.0	0.54	2.16	4.79	8.55
34	4	6	12	5	0.75	0.33	14.7	81.2	0.60	3.85	0.95	3.01
35	4	6	3	0	0.75	1.33	-0.1	54.9	0.36	4.49	1.04	4.10
36	4	6	3	2.5	-0.50	1.33	4.2	102.0	0.53	4.95	1.00	4.37
37	4	6	3	2.5	2.00	1.33	1.9	49.3	0.45	5.77	1.01	5.21
38	4	6	4.31805	2.5	0.75	0.93	2.9	51.2	0.35	2.72	1.07	2.59
39	4	6	7.5	0.73225	0.75	0.53	4.3	77.7	0.50	4.32	0.87	2.99
40	4	6	7.5	0	-0.50	0.53	3.2	96.0	0.48	4.93	1.35	5.22
41	4	6	7.5	0	2.00	0.53	3.5	85.1	0.59	4.07	1.05	3.54
42	4	6	7.5	2.5	-0.13	0.53	3.9	96.7	0.58	3.43	0.97	2.96
43	4	6	7.5	2.5	1.63	0.53	12.1	86.5	0.64	2.91	1.12	2.71
44	4	6	7.5	4.26775	0.75	0.53	5.9	77.0	0.52	4.51	0.95	3.76
45	4	6	7.5	5	-0.50	0.53	13.2	72.9	0.52	4.80	0.82	3.55
46	4	6	7.5	5	2.00	0.53	15.1	68.0	0.62	4.22	1.10	3.68
47	4	8.1213	7.5	2.5	0.75	0.53	5.9	99.8	0.61	2.34	0.95	1.84
48	4	9	12	2.5	0.75	0.33	4.4	119.2	0.37	1.65	0.92	1.25
49	4	9	3	2.5	0.75	1.33	2.0	79.8	0.16	4.62	1.01	3.91
50	4	9	7.5	0	0.75	0.53	2.1	116.7	0.43	2.40	0.85	1.79
51	4	9	7.5	2.5	-0.50	0.53	1.5	120.8	0.37	1.26	0.72	0.76
52	4	9	7.5	2.5	2.00	0.53	6.9	87.9	0.22	1.78	0.93	1.37
53	4	9	7.5	5	0.75	0.53	4.4	112.8	0.36	1.74	0.99	1.55
54	5	6	12	2.5	0.75	0.42	17.8	73.3	0.62	3.87	1.13	3.55
55	5	6	3	2.5	0.75	1.67	3.0	52.9	0.45	11.80	0.68	6.75
56	5	6	7.5	0	0.75	0.67	1.7	91.0	0.52	6.66	0.76	4.17
57	5	6	7.5	2.5	-0.50	0.67	7.8	95.4	0.63	6.32	0.79	4.17
58	5	6	7.5	2.5	2.00	0.67	12.9	84.4	0.54	5.28	0.78	3.75
59	5	6	7.5	5	0.75	0.67	16.2	78.3	0.61	6.27	0.85	4.58
60	5	9	7.5	2.5	0.75	0.67	2.5	131.4	0.30	3.49	1.04	2.62

61	3.0761	6	7.5	2.5	0.75	0.41	11.3	14.2	0.78	2.43	1.01	2.13
62	3.2929	6	7.5	2.5	-0.50	0.44	3.9	79.7	0.52	2.49	1.07	2.15
63	3.6173	6	7.5	2.5	0.75	0.48	8.6	81.9	0.59	2.61	0.96	2.24
64	3	4.5	7.5	2.5	-0.50	0.40	0.9	58.7	0.31	3.20	0.84	2.46
65	3	4.9393	7.5	2.5	0.75	0.40	9.4	3.3	0.80	2.93	0.94	2.33
66	3	6	12	0	0.75	0.25	4.6	76.1	0.50	2.71	1.08	2.44
67	3	6	12	2.5	-0.50	0.25	1.8	72.8	0.24	2.94	0.89	2.26
68	3	6	12	2.5	2.00	0.25	1.8	78.2	0.34	2.71	0.97	2.19
69	3	6	12	5	0.75	0.25	8.6	77.5	0.49	2.69	1.14	2.81
70	3	6	3	0	0.75	1.00	1.4	33.0	0.26	1.61	0.65	0.89
71	3	6	3	2.5	-0.50	1.00	2.1	52.2	0.38	1.43	0.53	0.63
72	3	6	3	2.5	2.00	1.00	1.9	51.1	0.35	1.06	0.66	0.61
73	3	6	3	5	0.75	1.00	3.5	51.7	0.45	2.26	0.75	1.54
74	3	6	7.5	0	-0.50	0.40	7.7	30.5	0.72	2.83	0.86	2.17
75	3	6	7.5	0	2.00	0.40	1.4	71.4	0.47	2.19	1.00	1.84
76	3	6	7.5	2.5	-0.13	0.40	2.2	76.5	0.54	3.06	0.95	2.45
77	3	6	7.5	5	2.00	0.40	6.6	15.2	0.79	1.89	1.19	2.15
78	3	7.0607	7.5	2.5	0.75	0.40	5.7	70.1	0.56	2.59	1.08	2.40
79	3	7.5	7.5	2.5	-0.50	0.40	-0.7	1.0	0.80	2.15	0.79	1.43
80	4.3827	6	7.5	2.5	0.75	0.58	15.2	77.9	0.69	4.12	1.00	3.73
81	4.7071	4.5	7.5	2.5	0.75	0.63	14.3	79.2	0.68	4.90	0.89	4.10
82	4.7071	6	12	2.5	0.75	0.39	11.4	90.0	0.45	4.81	0.84	3.67
83	4.7071	6	3	2.5	0.75	1.57	1.3	54.4	0.43	7.12	1.06	6.65
84	4.7071	6	7.5	5	0.75	0.63	9.2	82.9	0.51	4.61	0.99	3.94
85	4.7071	7.5	7.5	2.5	0.75	0.63	10.3	99.0	0.66	3.69	1.01	2.70
86	4.9239	6	7.5	2.5	0.75	0.66	12.2	93.8	0.62	6.09	0.75	3.66
87	4	4.5	10.682	2.5	0.75	0.37	8.3	61.8	0.46	4.76	0.86	3.47
88	4	4.5	12	0	0.75	0.33	-0.2	1.9	0.87	5.18	0.91	4.19
89	4	4.5	12	2.5	2.00	0.33	13.3	45.8	0.64	4.28	0.94	3.58
90	4	4.5	3	0	0.75	1.33	-0.4	36.3	0.26	4.07	1.08	3.95
91	4	4.5	3	2.5	2.00	1.33	2.3	51.5	0.25	3.44	0.88	2.41
92	4	4.5	7.5	0	2.00	0.53	3.0	77.8	0.55	5.89	0.86	4.40
93	4	4.5	7.5	4.2678	0.75	0.53	6.5	73.9	0.54	4.77	0.88	3.67

94	4	4.5	7.5	5	2.00	0.53	13.4	62.3	0.66	4.43	0.95	3.49
95	4	4.6142	7.5	2.5	0.75	0.53	14.2	68.2	0.67	3.45	0.94	2.90
96	4	4.9393	12	2.5	0.75	0.33	10.6	4.8	0.78	5.03	0.93	4.19
97	4	4.9393	3	2.5	0.75	1.33	0.3	37.7	0.26	4.20	1.03	3.44
98	4	4.9393	7.5	0	0.75	0.53	1.7	67.4	0.58	5.92	0.73	3.84
99	4	4.9393	7.5	2.5	-0.50	0.53	8.5	67.9	0.56	4.76	0.77	3.24
100	4	4.9393	7.5	2.5	2.00	0.53	11.9	54.9	0.61	5.00	0.94	4.29
101	4	4.9393	7.5	5	0.75	0.53	11.3	75.6	0.60	3.62	1.02	3.42
102	4	5.426	7.5	2.5	0.75	0.53	10.0	79.4	0.65	4.14	0.88	3.03
103	4	6	10.682	0	0.75	0.37	3.0	89.6	0.40	3.71	0.95	3.11
104	4	6	10.682	2.5	0.75	0.37	8.3	84.6	0.41	4.72	0.98	3.91
105	4	6	11.657	2.5	0.75	0.34	6.0	81.8	0.35	3.54	1.08	3.10
106	4	6	12	0.73223	0.75	0.33	11.0	12.2	0.77	3.86	0.84	2.81
107	4	6	12	0	-0.50	0.33	3.9	91.5	0.30	4.84	1.75	6.96
108	4	6	12	2.5	1.63	0.33	5.3	20.0	0.85	3.60	1.03	3.10
109	4	6	12	5	-0.50	0.33	7.7	0.7	0.70	3.83	1.00	3.46
110	4	6	12	5	2.00	0.33	10.8	93.6	0.42	4.74	1.12	4.12
111	4	6	3.3425	2.5	0.75	1.20	2.3	52.4	0.38	4.95	1.03	4.51
112	4	6	3	0.73223	0.75	1.33	1.1	53.7	0.48	4.77	0.99	4.24
113	4	6	3	0	-0.50	1.33	1.9	54.9	0.40	4.30	0.96	3.70
114	4	6	3	0	2.00	1.33	2.3	50.8	0.42	4.35	1.10	4.20
115	4	6	3	2.5	1.63	1.33	1.7	50.9	0.45	5.71	1.01	5.23
116	4	6	3	5	-0.50	1.33	2.5	52.2	0.33	2.68	0.94	2.30
117	4	6	3	5	2.00	1.33	2.7	49.5	0.40	2.39	1.18	2.24
118	4	6	4.318	0	0.75	0.93	2.9	101.3	0.83	3.08	1.01	2.77
119	4	6	4.318	2.5	0.75	0.93	0.8	52.0	0.25	2.98	1.06	2.08
120	4	6	5.7779	2.5	0.75	0.69	6.3	70.1	0.59	4.63	0.75	3.04
121	4	6	7.5	0.1903	0.75	0.53	3.0	60.8	0.25	3.81	1.12	3.60
122	4	6	7.5	0.73223	-0.50	0.53	2.7	95.8	0.51	4.68	0.82	3.48
123	4	6	7.5	0.73223	2.00	0.53	2.0	71.0	0.34	3.27	1.01	2.92
124	3	6	7.5	5	-0.50	0.40	6.0	74.9	0.53	2.78	0.90	2.19
125	4	4.5	7.5	0	-0.50	0.53	2.3	79.4	0.55	5.29	0.77	3.73
126	4	6	12	0	2.00	0.33	6.8	95.9	0.56	3.77	1.03	3.41

127	4	6	7.5	0	-0.13	0.53	4.1	80.9	0.45	4.70	0.85	3.57
128	4	7.0607	7.5	2.5	2.00	0.53	9.4	89.6	0.70	3.11	0.96	2.65
129	5	6	12	0	0.75	0.42	-1.0	-0.1	0.82	4.23	1.07	4.04
130	4	6	7.5	0	1.63	0.53	2.0	96.1	0.51	4.22	0.98	3.72
131	4	6	7.5	1.5433	0.75	0.53	9.4	74.4	0.57	2.74	1.04	2.47
132	4	6	7.5	2.5	-0.40	0.53	6.7	87.5	0.58	2.84	0.88	2.25
133	4	6	7.5	2.5	1.23	0.53	8.1	75.3	0.54	3.03	1.17	3.20
134	4	6	7.5	2.5	1.90	0.53	15.4	68.8	0.70	3.85	1.02	3.14
135	4	6	7.5	2.5	0.27	0.53	6.9	84.0	0.60	4.22	0.80	3.04
136	4	6	7.5	3.4567	0.75	0.53	13.4	68.7	0.67	4.55	0.87	3.23
137	4	6	7.5	4.2678	2.00	0.53	13.8	77.1	0.60	4.41	1.11	4.30
138	4	6	7.5	4.8097	0.75	0.53	6.5	84.4	0.50	3.25	0.98	2.76
139	4	6	7.5	5	1.63	0.53	10.0	81.9	0.51	3.06	1.09	3.12
140	4	6	9.2221	2.5	0.75	0.43	-0.9	0.9	0.74	4.75	0.90	3.68
141	4	6.574	7.5	2.5	0.75	0.53	15.0	61.0	0.70	3.61	0.96	3.12
142	4	7.0607	12	2.5	0.75	0.33	5.1	103.5	0.35	4.03	1.04	3.46
143	4	7.0607	3	2.5	0.75	1.33	1.2	54.8	0.37	5.74	1.00	5.07
144	4	7.0607	7.5	0	0.75	0.53	6.4	59.6	0.80	2.66	0.85	2.00
145	4	7.0607	7.5	2.5	-0.50	0.53	6.5	33.8	0.72	3.74	1.09	3.36
146	4	7.0607	7.5	5	0.75	0.53	6.9	89.8	0.54	3.84	0.83	2.68
147	4	7.3858	7.5	2.5	0.75	0.53	7.5	92.6	0.62	2.55	1.11	2.22
148	4	7.4	7.5	0	2.00	0.53	3.6	79.2	0.35	3.61	0.99	2.97
149	4	7.5	10.682	2.5	0.75	0.37	6.1	101.3	0.39	3.28	1.07	2.57
150	4	7.5	12	0	0.75	0.33	10.3	31.9	0.67	3.61	0.99	2.98
151	4	7.5	12	2.5	2.00	0.33	6.7	109.5	0.43	2.51	1.17	2.30
152	4	7.5	3	0	0.75	1.33	13.5	58.2	0.35	3.79	0.97	3.09
153	4	7.5	3	2.5	2.00	1.33	1.8	51.7	0.27	4.46	1.12	4.45
154	4	7.5	7.5	0	-0.50	0.53	1.5	102.8	0.41	3.58	0.89	2.74
155	4	7.5	7.5	4.2678	0.75	0.53	16.8	52.7	0.74	2.79	0.96	2.35
156	4	7.5	7.5	5	2.00	0.53	17.6	73.3	0.67	3.52	0.88	2.65
157	5	4.5	12	2.5	0.75	0.42	0.2	2.1	0.86	6.46	0.87	4.74
158	5	4.5	3	2.5	0.75	1.67	3.5	36.0	0.26	9.19	0.91	7.31
159	5	4.5	7.5	5	0.75	0.67	6.7	64.1	0.48	5.50	0.90	4.32

160	5	4.9393	7.5	2.5	0.75	0.67	14.2	48.2	0.82	5.20	0.83	4.06
161	5	6	10.682	2.5	0.75	0.47	6.4	94.1	0.46	3.09	0.97	2.77
162	5	6	12	2.5	-0.50	0.42	1.5	0.3	0.84	6.11	0.87	4.61
163	5	6	12	2.5	2.00	0.42	8.9	96.1	0.41	5.43	0.90	3.90
164	5	6	12	5	0.75	0.42	11.9	113.7	0.50	5.06	1.01	4.07
165	5	6	3	0	0.75	1.67	-0.6	34.4	0.26	10.17	0.76	6.67
166	5	6	3	2.5	-0.50	1.67	2.5	54.0	0.42	8.70	0.87	6.60
167	5	6	3	2.5	2.00	1.67	2.0	51.7	0.44	10.42	1.11	8.99
168	5	6	3	5	0.75	1.67	2.0	49.6	0.41	10.29	0.81	6.78
169	5	6	4.318	2.5	0.75	1.16	1.1	53.0	0.32	8.03	0.82	5.73
170	5	6	7.5	0	-0.50	0.67	1.8	89.8	0.43	5.44	0.77	3.47
171	5	6	7.5	0	2.00	0.67	11.8	31.3	0.81	4.52	0.95	3.84
172	5	6	7.5	4.2678	0.75	0.67	18.5	85.7	0.62	5.65	0.79	4.13
173	5	6	7.5	5	-0.50	0.67	17.7	92.1	0.62	5.29	0.84	3.63
174	5	6	7.5	5	2.00	0.67	18.5	44.4	0.77	5.83	0.82	4.11
175	5	7.0607	7.5	2.5	0.75	0.67	5.5	112.7	0.53	3.25	0.94	2.53
176	5	7.5	12	2.5	0.75	0.42	-1.8	0.0	0.75	4.57	1.07	4.08
177	5	7.5	3	2.5	0.75	1.67	13.9	55.1	0.38	10.11	0.88	7.85
178	5	7.5	7.5	5	0.75	0.67	14.0	97.3	0.56	3.32	0.99	3.04
